# Exploring the transformation of chemical components and the discovery of anti-tumor active components in the fruit of *Sinopodophyllum hexandrum*

**DOI:** 10.3389/fnut.2025.1555318

**Published:** 2025-03-31

**Authors:** Xiang-mu Tian, Shuai Wang, Tian-jiao Li, Xin-xin Yang, Yong-rui Bao, Xian-sheng Meng

**Affiliations:** ^1^College of Pharmacy, Liaoning University of Traditional Chinese Medicine, Dalian, China; ^2^Liaoning Multi-Dimensional Analysis of Traditional Chinese Medicine Technical Innovation Center, Dalian, China; ^3^Liaoning Province Modern Chinese Medicine Research Engineering Laboratory, Dalian, China; ^4^Shenyang Key Laboratory for Causes and Drug Discovery of Chronic Diseases, Shenyang, China

**Keywords:** fruit of *Sinopodophyllum hexandrum* (FSH), network pharmacology, composition analysis, anti-tumor, active ingredient

## Abstract

**Introduction:**

The fruit of *Sinopodophyllum hexandrum* (FSH) is derived from *Sinopodophyllum hexandrum* (Royle) Ying, a plant belonging to the family Berberidaceae of the order Ranunculaceae. It is mainly distributed in the Himalayan alpine region, and born in the understory of forests, and wetlands at the edge of forests, thickets or grasses. FSH grows at an altitude of 2,200–4,300 meters above sea level. Its main pharmacological activities include anti-tumor, anti-inflammation, analgesia, heat clearing and detoxification. In the current experiment, ultra-performance liquid chromatography quadrupole time-of-flight mass spectrometry (UPLC-Q-TOF-MS) was adopted for investigating the chemical components contained in FSH, their transformation patterns *in vivo* and the potential anti-tumor components, so as to provide an experimental basis for the utilization and development of the resources of FSH.

**Methods:**

The chemical components of FSH and their transformation patterns *in vivo* were investigated by UPLC-Q-TOF-MS, and the potential anti-tumor active components were predicted from the *in vivo* transformed components of FSH by using a network pharmacology approach.

**Results:**

Totally 85 chemical components were identified in FSH, among which, 61 were flavonoids and 24 were lignans. The above components were transformed *in vivo*, including 36 prototype components and 13 transformed products. As revealed by the results of network pharmacology on the prediction of anti-tumor components of FSH, 17 compounds such as Kaempferol, Uralenol, and 8-Prenylquercetin in FSH were used as the potential anti-tumor components.

**Conclusion:**

In this study, the chemical composition, *in vivo* transformed components of FSH and their metabolites are investigated, and the *in vivo* transformed components are predicted to have potential anti-tumor pharmacological activities. This study provides the experimental bases for the utilization and development of the resources of FSH.

## 1 Introduction

The fruit of *Sinopodophyllum hexandrum* (FSH) is derived from *Sinopodophyllum hexandrum* (Royle) Ying, a plant belonging to the Berberidaceae family. First officially included in the 2010 edition of the “Chinese Pharmacopoeia” (1), FSH is a perennial herb with a height of 20–50 cm. Its rhizome is short, stout, and nodding, with many fibrous roots, whereas the stem is erect, simple, longitudinal, and glabrous, and the base is covered with large brown scales. Its leaves are thinly papery, peltate, base cordate, glabrous, and pilose abaxially, with a coarsely serrate margin. Its petioles are longitudinally ribbed and glabrous. Moreover, the flowers are large, solitary, pink, bisexual and open before leaves. It has 6 sepals, 6 petals, 6 stamens, and the filaments are slightly shorter than anthers, which are linear, longitudinally lobed, and apex rounded, with non-extended connectives. There is one pistil, the ovary is ellipsoid, with one locule and laterally membranous placentation, which contained numerous ovules, style short, and stigma capitate. The berry is ovoid, with the length of 4–7 cm, the diameter of 2.5–4 cm, and is orange-red when ripe. Additionally, the seeds are ovate-triangular and brownish red, without fleshy aril. The flowering period is from May to June, and the fruiting period is from July and September (2). *Sinopodophyllum hexandrum* is mainly distributed in the Himalayan alpine region, including China, India, Nepal, Pakistan and the nearby understory, forest edge wetland, scrub or grassland and other cold and wet areas at an altitude of 2,200–4,300 m. In China, it is mainly distributed in Xizang, Sichuan and Yunnan.

FSH has long been used in Tibetans. At present, there are many reports that FSH has health care functions, and it has been developed into health care wine, health care tea and other products. Local people have the habit of picking and eating this fruit directly (3). Furthermore, as documented in the Jingzhu Bencao, FSH can be used to treat venous disease and uterine disease (4). Besides, it is recorded in the Ruyi Baoshu that, FSH has a special effect on the treatment of blood disease and gynecological disease (4). According to modern pharmacology, FSH exhibits anti-tumor, anti-viral, anti-microbial, immunomodulatory, and anti-inflammatory effects (5, 6).

Existing studies on the chemical composition of FSH have proved that FSH mainly contains flavonoids and lignans (4, 7–10), of which, flavonoids mainly comprise quercetin-3-methyl ether-3′/4’-O-glucoside, citrusinol, 6′-prenylquercetin-3-methyl ether, quercetin and other compounds, whereas lignans mostly encompass podophyllotoxne isomer, demethyldesoxypo-dophyllotoxin isomer diglucoside, *α*-peltatin or isomer, desoxypodophyllotoxin and other compounds. Based on a comprehensive review of the relevant literature, flavonoids and lignans present in FSH exhibit inhibitory effects on various tumor cell lines (6) and demonstrate significant anti-tumor activity (4, 11). Among the lignans, podophyllotoxin and its derivatives, such as etoposide and teniposide, are extensively utilized as anti-tumor agents in clinical settings (9, 10, 12). The mechanism underlying their action is associated with the modulation of the PI3K/AKT/mTOR signaling pathway, leading to the induction of apoptosis and autophagy in tumor cells (13). Furthermore, podophyllotoxin exhibits anti-herpes simplex virus and immunosuppressive properties, demonstrating significant efficacy in the clinical management of HPV. Flavonoids in FSH also possess antibacterial, anti-inflammatory, and immunomodulatory activities, making them applicable in the treatment of hepatitis B and bronchitis. These pharmacological properties not only underpin the use of FSH in traditional Tibetan medicine but also offer promising avenues for the development of modern therapeutics, particularly highlighting its potential in anti-tumor clinical applications.

Currently, relevant studies have reported the chemical components of FSH, but the process of how these components are transformed in organisms has not been reported. And there are also few reports on which components have potential anti-tumor activities. To solve the above problems of FSH, this experiment first identified 87 chemical components of FSH by ultra-performance liquid chromatography quadrupole time-of-flight mass spectrometry (UPLC-Q-TOF-MS), and subsequently explored the transformation process of these chemical components *in vivo*. Meanwhile, combined with the network pharmacology approach, 17 potential anti-tumor active components were identified among the transformed chemical components *in vivo*. The above results offer a preliminary basis for the development and utilization of FSH plants and the research and development of new anti-tumor drugs.

## 2 Materials and methods

### 2.1 Chemicals and reagents

FSH samples were harvested from five random locations within the range of Chamdo Lado Township (S1: longitude E: 97°40′, latitude N: 31°50′; S2: longitude E: 97°41′, latitude N: 31°56′; S3: longitude E: 97°40′, latitude N: 31°64′; S4: longitude E: 97°34′, latitude N: 31°55′; S5: longitude E: 97°45′, latitude N: 31°54′) in the Tibet Autonomous Region, and dried in the shade after harvesting. All FSH samples were identified as mature fruit of *Sinopodophyllum hexandrum* (Royle) Ying by Prof. Zhang Jiankui from Liaoning University of Traditional Chinese Medicine. Quercetin-3-O-glucoside, kaempferol-3-O-rutinoside, quercetin-3-methyl ether, uralenol, podophyllotoxne and podophyllotoxin control products (purity≥98%, Baoji Weibin District Xinyi Scientific Instruments Business Department), methanol (MERCK, Germany; batch no. l1143835114), acetonitrile (MERCK, Germany; batch no. l1165729132), formic acid (Thermo Fisher Scientific (China) Co., Ltd.; batch no. 214911), and sodium heparin were also utilized in this experiment. DMEM, L-15 and 1,640 medium (Dalian Meilun Biotechnology Co., Ltd., China), human hepatocellular carcinoma cells (HepG2) (Qi’s (Shanghai) Biotechnology Co., Ltd., China), human colon cancer cells (SW620) (Saibaikang Biotechnology Co., Ltd., China), human non-small cell lung cancer cells (A549) and mouse mammary carcinoma cells (4T1) (Cell Bank of the Chinese Academy of Sciences, China).

### 2.2 Thin layer chromatography identification of FSH

In accordance with the method of “Identification (2)” of FSH under the item of “Medicinal Materials and Drinking Tablets” in Part I of the 2020 edition of the Chinese Pharmacopoeia. FSH samples collected from five locations were identified by TLC, so as to determine the correct source of the fruit. The specific experimental process is as follows: accurately weigh 5.00 g of FSH powder, then accurately add 10 mL of methanol and perform ultrasonic extraction at 40 kHz for 20 min. After extraction, filter the solution and dry the filtrate, add 2 mL of methanol to dissolve the residue, which will serve as the FSH sample solution. A methanol solution of podophyllotoxin with a concentration of 0.5 mg∙mL^−1^ is used as the control solution. Pipette 4 μL of each of the above test solutions and control solutions onto the same silica gel G thin-layer plate. Use a mixture the upper solution of cyclohexane, water-saturated n-butanol, and formic acid (6.5, 2.5, 0.8) as the unfolding agent. Allow the solutions to unfold and dry, then spray with a 1% vanillin sulfuric acid solution, and heat until the spots are clear.

### 2.3 Preparation of the decoction for UPLC-Q-TOF-MS analysis and oral administration

First of all, 2.00 g of the powdered FSH (Sample S1-S5: passed through the No.4 sieve) was weighed precisely, then 50 mL of methanol was added precisely for 30 min of sonication at 40 KHz, and the resultant sample was later passed through the 0.22 μm microporous filtration membrane to acquire the test solution. Thereafter, the obtained test solution was analyzed by UPLC-Q-TOF-MS and the results were imported into MassHunter Qualitative Analysis B.06.00 software for analysis and processing.

Approximately 30 g of the powdered FSH (Sample S1: passed through the No.4 sieve) was weighed, later 10 times the amount of water was supplemented to soak the sample for 15 min. Later, the resultant sample was heated and reflux extracted twice for 1 h each. After filtration, the two filtrates were combined and concentrated under the reduced pressure to a solution with a concentration of 1.0125 g mL^−1^ (with 1 mL solution containing 1.0125 g of the raw drug), thus the FSH solution of water was obtained for oral administration.

### 2.4 Animals handing and plasma samples preparation

Twelve SPF-grade healthy male SD rats weighing (200 ± 20) g (Liaoning Changsheng Biotechnology Co., Ltd.; Certificate of Conformity No.: SCXK (Liao) 2020-0001) were used in this experiment. Our experimental protocol was approved by the Ethics Committee of Liaoning University of Traditional Chinese Medicine (no. 2020059).

Altogether 12 healthy male SD rats (200 ± 20 g) were taken and randomly classified into the blank group and the drug administration group (*n* = 6 each). These rats were raised in an environment at the temperature of (25 ± 2)°C and relative humidity of (55 ± 5)%, under the natural day and night condition, with free access to water and standard maintenance feed (Liaoning Changsheng Biotechnology Co., Ltd.). The experiment was initiated after 1 week of adaptive feeding.

The rats in each group were weighed before the experiment, and after fasting without water for 12 h, the drug group was given FSH extract at 4.86 g∙Kg^−1^ by gavage according to 6 times the highest adult clinical dosage recommended by the Pharmacopoeia. Meanwhile, the rats in the blank group were given an equal amount of distilled water by gavage. On the third day of gavage, blood was collected from the orbital venous plexus of rats into the 2 mL centrifuge tube containing sodium heparin, slightly shaken, and allowed to stand at 4°C for 30 min. Then, the blood sample was subject to centrifugation at 4°C for 15 min at 3000 r∙min^−1^, and the supernatant was aspirated to obtain the plasma of FSH.

By optimizing the plasma sample preparation method of rats in the FSH administration group (14), the optimal plasma sample preparation process was shown as follows. In brief, 100 μL of plasma sample was collected from the FSH administration group, then 300 μL of the pre-cooled methanol was added at the plasma: solvent ratio of 1:3, and the resultant sample was subject to centrifugation at 4°C for 15 min at 12,000 r·min^−1^. Afterwards, the supernatant of the plasma sample was obtained after centrifugation, and then blown dried by the nitrogen gas. The residue was reconstituted by adding 50 μL of the pre-cooled methanol at the plasma: solvent ratio of 1:0.5, vortexed at 3000 r∙min^−1^ for 2 min, centrifuged at 4°C and 12,000 r∙min^−1^ for 15 min, and the supernatant was aspirated to obtain the plasma test solution of FSH. The plasma of rats in the blank group was treated in an identical way.

### 2.5 Instrumentation and chromatographic conditions

#### 2.5.1 Ultra performance liquid chromatography

The Agilent poroshell 120 SB-C18 column (100 mm × 4.6 mm, 2.7 μm) was applied in UPLC. The mobile phases were consisted of A water (0.1% formic acid) and B methanol with gradient elution (0 ~ 30 min, 5% ~ 100% B) in the positive ionization mode; whereas A water and B acetonitrile with gradient elution (0 ~ 30 min, 5% ~ 100% B) in the negative ionization mode. The injection volume in both modes was 2 μL, the flow rate was 0.4 mL∙min^−1^, the equilibrium column time was 8 min, and the column oven temperature was 30°C.

#### 2.5.2 Mass spectrometry

The electrospray ion source was used. In the positive ion mode, the capillary voltage was 4,000 V, sheath gas temperature was 350°C, dry gas temperature was 250°C, sheath gas flow rate was 11 L/min, dry gas flow rate was 13 L/min, nebulizer pressure was 45 psig, fragmentation voltage was 125 V, and mass range was 50 ~ 1,500 m/z. In the negative ion mode, all the MS conditions were the same as those in the positive ion mode, except for the capillary voltage of 3,500 V.

### 2.6 Data analysis

Through a review of the literature pertaining to the chemical composition of FSH, a comprehensive chemical composition information library was constructed using PCDL Manager B.07.00 software. This library was subsequently imported into MassHunter Qualitative Analysis B.06.00 software to facilitate the search for potential compounds, employing +H and + Na as positive adducts and -H and -COOH as negative adducts. The retention times and precise molecular weights of pertinent compounds were determined by analyzing the FSH mass spectra in both ionization modes. Secondary mass spectrometry analysis of the target compounds was conducted to detect their chemical compositions, utilizing retention time, mass-to-charge ratio, and fragmentation ion data from the secondary mass spectra. This information was then compared and analyzed against control products and relevant literature to complete the identification of the compounds. For the identified compounds, an allowable mass error of 10 ppm was established (15, 16).

Based on the identified *in vitro* chemical composition of FSH, a comprehensive database detailing the chemical composition of FSH and its potential *in vivo* metabolites was established, including products of oxidation, glucuronidation, and other reactions involving the prototype components. Utilizing this database, a comparative analysis of plasma mass spectra from both blank and FSH-administered rat groups was conducted. Compounds detected in the plasma of the administered group, but absent in the blank group, were further analyzed using MS/MS secondary mass spectrometry. The *in vivo* transformation of FSH constituents was inferred from retention times, secondary mass spectral fragmentation patterns, and additional pertinent data (17, 18).

### 2.7 Network pharmacology analysis

#### 2.7.1 Search for the main chemical components and target sites of FSH

Based on the above *in vivo* analysis results of the absorbed and transformed components of FSH, the derived chemical components were adopted to obtain the Canonical SMILES of the chemical components through PubChem.^1^ Besides, the Swiss target prediction^2^ was used for predicting the targets of pharmacodynamic components.

#### 2.7.2 Acquisition and screening of tumor-related targets

The GeneCards database^3^ and OMIM database^4^ were searched with the keyword “tumor,” and the disease-related targets were obtained after de-emphasis.

#### 2.7.3 Intersection of FSH-and tumor-related targets

The above disease targets were intersected with the screened gene targets for the roles of the absorbed and transformed components of FSH in tumor treatment. Venn diagrams were generated using the microbiological letter platform.

#### 2.7.4 Construction of the drug-component-target-disease network

To create a drug-component-target-disease network model, the active components of FSH and the gene targets and targets of FSH for tumor treatment were imported into Cytoscape 3.9.1 software.

#### 2.7.5 Construction of the protein–protein interaction network and screening of core targets

The intersected targets of FSH for tumor treatment were imported into the STRING database to construct the PPI network. After removing the free nodes, the network topology analysis was performed using Cytoscape 3.9.1 software. Thereafter, the topological parameter analysis of the network nodes was implemented with the Network Analyzer plug-in. Using the three parameters of betweenness centrality (BC), closeness centrality (CC) and degree value (degree) as indicators, the medians of the three indicators were calculated, respectively. When the target indicator was greater than the corresponding median, it was predicted as the core target. Finally, core targets were ranked in accordance with the degree value (degree).

#### 2.7.6 Gene ontology functional annotation and Kyoto encyclopedia of genes and genomes pathway enrichment analysis

GO and KEGG enrichment analyses were performed on the potential targets using the DAVID 6.8 database, with the species being defined as “*Homo Sapiens*,” and the screening criterion being set to *p* ≤ 0.05. The results of the top 10 GO and 25 KEGG enrichment analyses meeting the criteria were visualized in the bar charts.

### 2.8 Anti-tumor efficacy of chemical components in FSH

#### 2.8.1 Tumor cells and drug delivery components selection

Based on the screening results of the core components in network pharmacology, there were 10 chemical components, including 6’-Prenylquercetin-3-methyl ether, Uralenol, Kaempferol-4′-methyl ether, Kaempferol and so on, each exhibiting a Degree value of ≥40. Among these core constituents, Quercetin-3-methyl ether and others were only detected as metabolites in rat plasma, which led to their exclusion from further analysis. Consequently, Uralenol and Kaempferol were selected for validation of cellular efficacy. Following the classification of KEGG pathway screening results, tumor-related pathways were prioritized, encompassing prostate cancer, gastric cancer, non-small cell lung cancer, hepatocellular carcinoma, breast cancer, colorectal cancer, among others. Considering the pathways through which Uralenol and Kaempferol both exert their effects, human liver cancer cells (HepG2), human non-small cell lung cancer cells (A549), human colon cancer cells (SW620), and mouse mammary carcinoma cells (4T1) were ultimately selected for the cellular assays. For the selection of positive control drugs, Adriamycin, which is widely used in the treatment of hepatocellular carcinoma, breast cancer, and non-small cell lung cancer, and 5-fluorouracil, commonly used for colon cancer, were chosen as the positive controls for the cell tests. These drugs were used to demonstrate the inhibitory effects of the aforementioned components on the growth of different tumor cells.

#### 2.8.2 Anti-tumor efficacy evaluation

Different tumor cells were routinely cultured in 5% CO_2_ and 37°C. And HepG2 cells were cultured in DMEM, SW620 in L-15, and 4T1 and A549 in 1640 medium. Logarithmic-phase tumor cells were seeded at 1 × 10^5^ cells/mL in 96-well plates, 100 μL per well, and cultured for 12 h post-adhesion. Set up administration groups with different concentrations. HepG2 cells received Uralenol and Kaempferol (1, 10, 100, 200, 300 μmol/L) and 1 μmol/L doxorubicin; SW620 cells were treated with Kaempferol (1, 10, 100, 200, 300 μmol/L), Uralenol (1, 25, 50, 75, 100 μmol/L), and 50 μmol/L 5-fluorouracil; A549 cells received Kaempferol (1, 10, 100, 200, 300 μmol/L), Uralenol (1, 25, 50, 100, 200 μmol/L), and 1 μmol/L doxorubicin; 4T1 cells were given Uralenol and Kaempferol (1, 10, 100, 200, 300 μmol/L) and 5 μmol/L doxorubicin. The control group of all the cells mentioned above was treated with equal volumes of complete culture medium corresponding to the cells. After 24 h of administration, CCK-8 assay was used to measure the absorbance (OD) at 450 nm using an enzyme labeling instrument. The proliferation inhibition rate of the administered monomer on different tumor cells was calculated [cell proliferation inhibition rate = (1-OD value of the treated group cells/OD value of the control group cells) × 100%], as well as the IC50 value.

## 3 Results

### 3.1 TLC results

**Figure 1:**
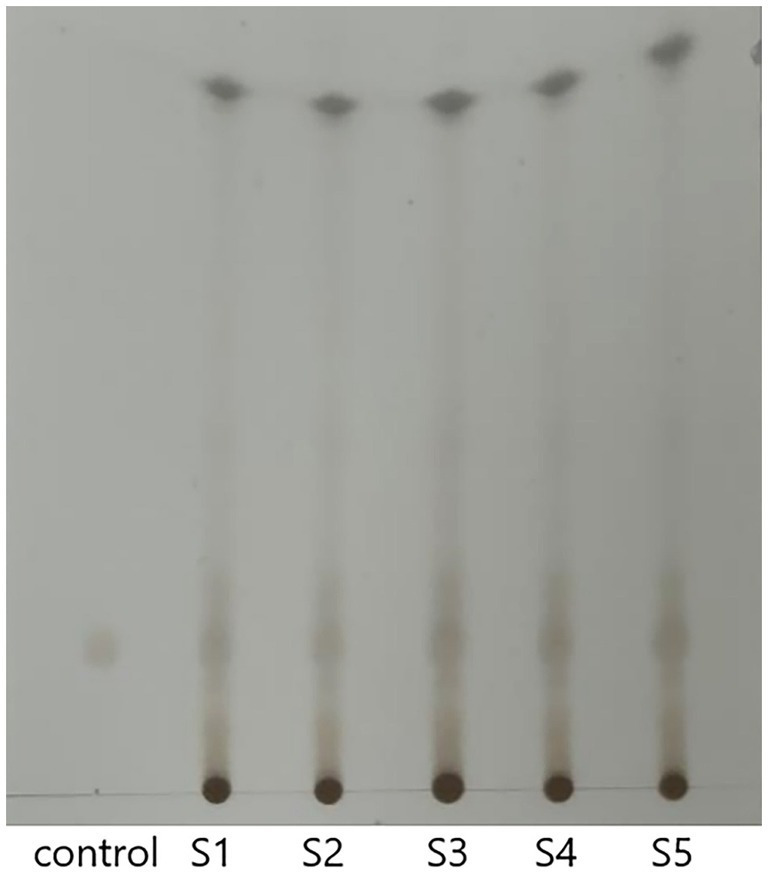
TLC results of FSH at different locations. *Control: Podophyllotoxin control solution; S1-S5: FSH samples from different locations.

The TLC results are presented in Figure 1. According to the thin-layer chromatographic identification method of FSH in the Chinese Pharmacopoeia, it can be observed that after the color development was complete, the test solution (FSH) and the control solution (Podophyllotoxin) exhibited the same color spots at the same position on the same silica gel plate. The Rf values for the different FSH samples were calculated as follows: Rf_control_: 0.224; Rf_S1_: 0.224; Rf_S2_: 0.224; Rf_S3_: 0.224; Rf_S4_: 0.224; Rf_S5_: 0.224 (calculated as: Specific Shift Value (Rf) = Distance traveled by the solute / Distance traveled by the solvent front). Suggesting that the above FSH samples were authentic according to the Pharmacopoeia testing methods. S1-S5 represent five different samples.

### 3.2 Analysis on the chemical composition and cleavage pattern of some compounds of FSH

**Figure 2:**
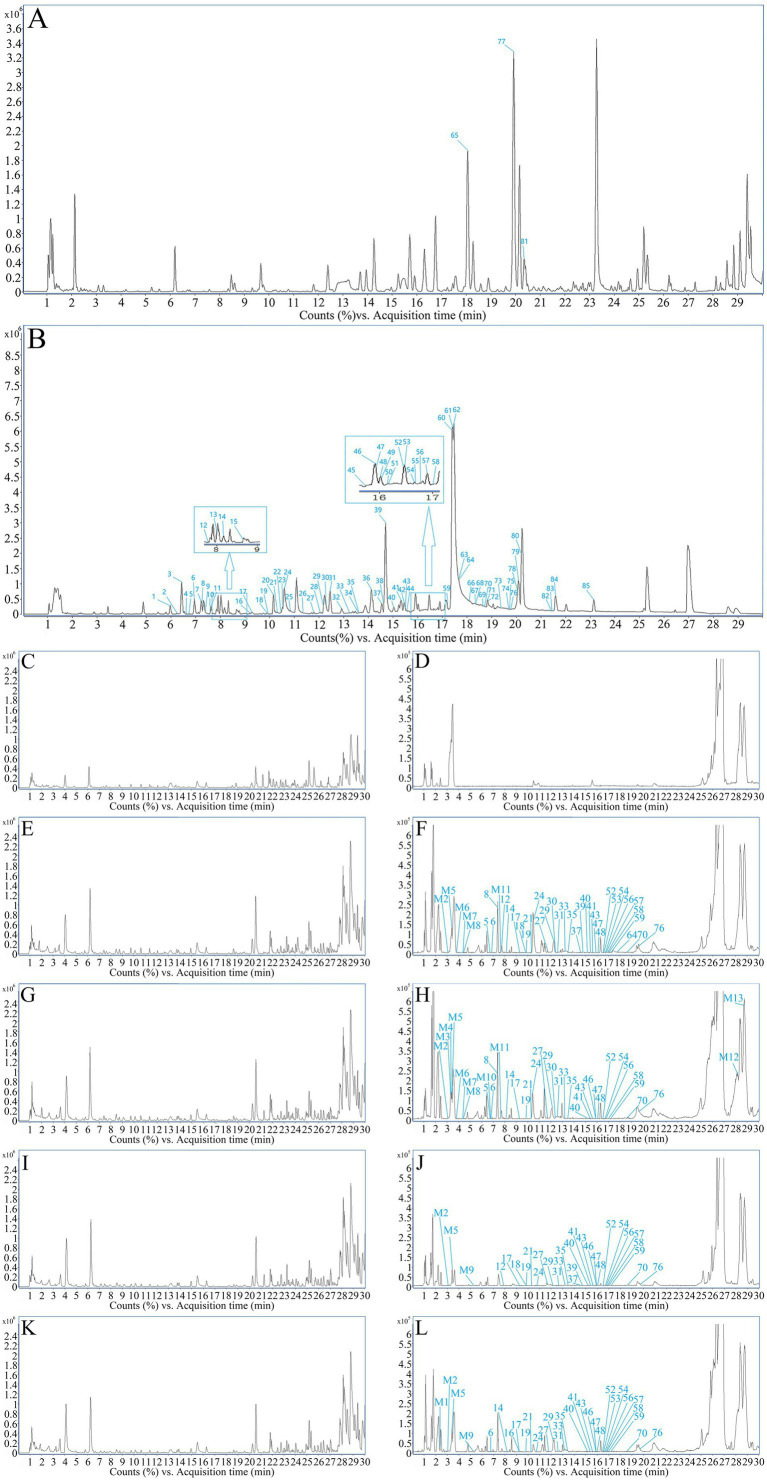
TIC profiles of FSH. **(A)** TIC profile in positive ion mode. **(B)** TIC profile in negative ion mode. **(C)** Positive ions in blank group rats. **(D)** Negative ions in blank group rats. **(E)** Positive ions in treatment group rats for 30 min. **(F)** Negative ions in treatment group rats for 30 min. **(G)** Positive ions in treatment group rats for 1 h. **(H)** Negative ions in treatment group rats for 1 h. **(I)** Positive ions in treatment group rats for 2 h. **(J)** Negative ions in treatment group rats for 2 h. **(K)** Positive ions in treatment group rats for 3 h. **(L)** Negative ions in treatment group rats for 3 h.

Through UPLC-Q-TOF-MS analysis, it was found that the chemical components present in the five samples of FSH (S1-S5) showed no differences in their types, except for slight variations in mass spectrometry response values. Therefore, sample S1 was selected as an example for further explanation. Based on the retention time of each chemical component in the FSH extract, MS data, extracted total ion-flow diagrams, and relevant literature reports, the chemical composition of the FSH test solution was analyzed, as shown in Figure 2 and Table 1. A total of 85 chemical components were obtained.

**Table 1:** Results of the chemical composition of FSH.

No.	t_R_/min	Measured value (m/z)	Theoretical value (m/z)	ppm	Fragment (m/z)	Molecular formula	Chemical compound	Ionization mode	*In vivo* conversion or not
1^a^	5.976	369.0979	369.0980	−0.27	247.0683, 220.0716, 219.0637, 175.0828	C_20_H_18_O_7_	gancaonin P (23)	[M-H]^−^	-
2^a^	6.242	625.1397	625.1410	−2.08	464.4814, 463.0792, 302.0378, 301.0340	C_27_H_30_O_17_	quercetin-3,7-di-O-*β*-D-glucoside (23)	[M-H]^−^	-
3^a^	6.449	479.0813	479.0831	−3.76	316.0247, 271.0720, 214.8611	C_21_H_20_O_13_	myricetin-3-O-glucoside (38)	[M-H]^−^	-
4^a^	6.626	533.1652	533.1664	−2.25	371.1191, 356.6958, 341.4304, 257.9164	C_26_H_30_O_12_	kaempferol,8-(3-hydroxy-3-methylbutyl)-7-glucoside (23)	[M-H]^−^	-
5^a^	6.838	477.1053	477.1038	3.14	316.0548, 315.0523, 300.0276, 271.0252, 255.0227	C_22_H_22_O_12_	quercetin-3-methyl ether-3′/4’-O-glucoside (19)	[M-H]^−^	√
6^a^	6.981	609.1458	609.1461	−0.49	343.0441, 301.0344, 271.0240, 255.0352, 151.0058	C_27_H_30_O_16_	rutin (20)	[M-H]^−^	√
7^a^	7.335	463.0891	463.0882	1.94	301.0318, 300.0287, 271.0301, 178.9964, 151.0055	C_21_H_20_O_12_	quercetin-7-O-glucoside (4, 7)	[M-H]^−^	-
8^a*^	7.363	463.0872	463.0882	−2.16	301.0326, 300.0278, 271.0282, 255.0291	C_21_H_20_O_12_	quercetin-3-O-glucoside (4, 7)	[M-H]^−^	√
9^b^	7.549	607.1655	607.1668	−2.14	561.0267, 399.1068, 397.1258, 381.0992, 307.1403, 323.0176	C_27_H_30_O_13_	4′-demethylepipodophyllotoxin glucoside (20)	[M + HCOO]^−^	-
10^a^^*^	7.637	593.1507	593.1512	−0.84	286.0446, 285.0405, 255.0224, 229.6993, 211.0364	C_27_H_30_O_15_	kaempferol-3-O-rutinoside (39)	[M-H]^−^	-
11^a^	7.795	369.0971	369.0980	−2.44	235.0603, 283.0694, 178.9958	C_20_H_18_O_7_	A-ring-monohydroxylated-8-prenylkaempferol (39)	[M-H]^−^	-
12^a^	7.883	477.1024	477.1038	−2.93	315.0520, 300.0277, 271.0185	C_22_H_22_O_12_	quercetin-3-methyl ether-3′/4’-O-glucoside (19)	[M-H]^−^	√
13^a^	8.057	447.0929	447.0933	−0.89	285.0382, 284.0326, 255.0285, 229.0509, 163.0036	C_21_H_20_O_11_	kaempferol-3-O-glucoside (23)	[M-H]^−^	-
14^b^	8.179	399.1077	399.1085	−2.00	384.0878, 314.0450, 284.0400, 257.0464, 241.0428	C_21_H_20_O_8_	α-peltatin or isomer (40)	[M-H]^−^	√
15^b^	8.771	707.2173	707.2193	−2.83	545.9067, 383.1150, 325.0169, 179.0004, 151.0049	C_33_H_40_O_17_	demethyldesoxypo-dophyllotoxin isomer diglucoside (23)	[M-H]^−^	-
16^a^	9.223	477.1045	477.1038	1.47	315.0495, 300.0226, 299.0172	C_22_H_22_O_12_	quercetin-3-methyl ether-7-O-glucoside (19)	[M-H]^−^	√
17^b^	9.288	399.1088	399.1085	0.75	383.0747, 366.0762, 355.0910, 340.0970, 270.0442	C_21_H_20_O_8_	α-peltatin or isomer (40)	[M-H]^−^	√
18^a^	9.917	367.0810	367.0823	−3.54	352.0520, 293.0688, 266.0580, 193.0146	C_20_H_16_O_7_	2′,3′-(2″,2″-dimethylpyrane)-3,7,5,4′-tetrahydroxy flavone or isomer (4)	[M-H]^−^	√
19^b^	9.963	399.1080	399.1085	−1.25	384.0862, 355.0810, 399.0855, 329.0855, 314.0440	C_21_H_20_O_8_	α-peltatin or isomer (40)	[M-H]^−^	√
20^b^	10.176	621.1823	621.1825	−0.32	575.1790, 413.1256, 383.1156, 355.1458, 189.6925	C_28_H_32_O_13_	β-peltatin-O-β-D-glucoside (23)	[M + HCOO]^−^	-
21^b^	10.263	399.1090	399.1085	1.25	383.0758, 369.0643, 355.0809, 341.0674	C_21_H_20_O_8_	α-peltatin or isomer (40)	[M-H]^−^	√
22^a^	10.484	301.0351	301.0354	−1.00	193.0159, 179.0009, 151.0039	C_15_H_10_O_7_	Quercetin (4)	[M-H]^−^	-
23^b^	10.545	399.1079	399.1085	−1.50	384.0868, 339.0840, 297.0416, 279.0353, 175.0056	C_21_H_20_O_8_	α-peltatin or isomer (40)	[M-H]^−^	-
24^b^	10.761	399.1085	399.1085	0.00	384.0862, 369.0624, 325.0715, 298.0438	C_21_H_20_O_8_	α-peltatin or isomer (40)	[M-H]^−^	√
25^a*^	10.928	315.0502	315.0510	−2.54	300.0293, 271.0288, 255.0311, 227.0365	C_16_H_12_O_7_	quercetin-3-methyl ether (21)	[M-H]^−^	-
26^b^	11.483	399.1121	399.1085	9.02	384.0858, 312.0275, 284.0304, 240.0414	C_21_H_20_O_8_	α-peltatin or isomer (40)	[M-H]^−^	-
27^b^	11.878	399.1103	399.1085	4.51	384.0860, 356.0945, 173.0630, 159.0491	C_21_H_20_O_8_	α-peltatin or isomer (40)	[M-H]^−^	√
28^b^	12.039	399.1101	399.1085	4.01	384.0464, 314.0443, 285.0436	C_21_H_20_O_8_	α-peltatin or isomer (40)	[M-H]^−^	-
29^b^	12.131	399.1097	399.1085	3.01	382.1045, 381.0979, 369.0942, 178.9998, 152.0114	C_21_H_20_O_8_	α-peltatin or isomer (40)	[M-H]^−^	√
30^a^	12.393	285.0407	285.0405	0.70	239.0342, 229.0549, 187.0479, 185.0589, 163.0022, 151.0040	C_15_H_10_O_6_	kaempferol (4)	[M-H]^−^	√
31^b^	12.599	399.1104	399.1085	4.76	384.0860, 312.0249, 285.0436, 283.0211	C_21_H_20_O_8_	α-peltatin or isomer (40)	[M-H]^−^	√
32^b^	12.930	399.1099	399.1085	3.51	384.0858, 367.0857, 284.0304, 179.0015	C_21_H_20_O_8_	α-peltatin or isomer (40)	[M-H]^−^	-
33^b^	13.250	399.1096	399.1085	2.76	339.0872, 323.0926, 281.0429, 233.0448	C_21_H_20_O_8_	α-peltatin or isomer (40)	[M-H]^−^	√
34^a^	13.675	367.0826	367.0823	0.82	323.0190, 312.0269, 295.0289, 284.0312, 255.0276, 251.0338	C_20_H_16_O_7_	7,8-(2″,2″-dimethyl pyrane)-3,5,3′,4′-tetrahydroxy flavone or isomer (4)	[M-H]^−^	-
35^b^	13.699	399.1094	399.1085	2.26	381.0995, 367.0857, 325.0345, 178.9966	C_21_H_20_O_8_	α-peltatin or isomer (40)	[M-H]^−^	√
36^a^	14.255	469.1845	469.1868	−4.90	395.1165, 265.0747, 247.0620, 219.0690	C_26_H_30_O_8_	Sinoflavonoids K (4)	[M-H]^−^	-
37^a*^	14.600	369.0967	369.0980	−3.52	351.0850, 323.0866, 299.0530, 255.0265	C_20_H_18_O_7_	Uralenol (49)	[M-H]^−^	√
38^a^	14.720	383.1128	383.1136	−2.09	368.0906, 327.0416, 284.0334, 267.0308, 256.0385	C_21_H_20_O_7_	2′-prenylquercetin-3-methyl ether (41)	[M-H]^−^	-
39^a^	14.875	369.0949	369.0980	−8.40	313.0386, 247.0572, 219.0688	C_20_H_18_O_7_	8-prenylquercetin (4)	[M-H]^−^	√
40^a^	15.084	383.1150	383.1136	3.65	368.0916, 369.0947, 326.0401, 314.0385, 176.0136	C_21_H_20_O_7_	6-prenylquercetin-3-methyl ether (4)	[M-H]^−^	√
41^a^	15.309	467.1705	467.1711	−1.28	449.1621, 437.1601, 247.0621, 219.0667	C_26_H_28_O_8_	dysosmaflavone F (4)	[M-H]^−^	√
42^a^	15.582	453.1554	453.1555	−0.22	435.1427, 383.1177, 247.0614, 219.0635, 175.0733	C_25_H_26_O_8_	8-prenyl-3′,4′-(2″,2″-dimethyl-4″-hydroxy-3″,4″-dihydropyran-e)-3,5,7,4′-tetrahydroxy flavone or isomer (4)	[M-H]^−^	-
43^a^	15.670	299.0555	299.0561	−2.01	284.0350, 255.0308, 227.0363, 183.0473, 167.0518	C_16_H_12_O_6_	kaempferol-3-methyl ether (23)	[M-H]^−^	√
44^a^	15.777	367.0823	367.0823	0.00	311.0915, 173.0600, 131.0505	C_20_H_16_O_7_	7,8-(2″,2″-dimethyl pyrane)-3,5,3′,4′-tetrahydroxy flavone or isomer (4)	[M-H]^−^	-
45^a^	15.811	453.1549	453.1555	−1.32	435.1427, 395.1150, 367.1198, 351.1219, 247.0630, 219.0688	C_25_H_26_O_8_	8-prenyl-3′,4′-(2″,2″-dimethyl-4″-hydroxy-3″,4″-dihydropyran-e)-3,5,7,4′-tetrahydroxy flavone or isomer (4)	[M-H]^−^	-
46^a^	15.944	299.0562	299.0561	0.33	284.0365, 227.0364, 164.0098, 151.0046	C_16_H_12_O_6_	kaempferol-4′-methyl ether (22)	[M-H]^−^	√
47^a^	15.961	383.1156	383.1136	5.22	369.0950, 368.0913, 339.0899, 323.0925, 284.0276, 283.0244	C_21_H_20_O_7_	6′-prenylquercetin-3-methyl ether (4)	[M-H]^−^	√
48^a^	16.025	383.1152	383.1136	4.18	368.0917, 325.0364, 284.0354, 175.0053	C_21_H_20_O_7_	8-prenylquercetin-3-methyl ether (4)	[M-H]^−^	√
49^a^	16.072	451.1771	451.1762	1.99	451.1797, 247.0630, 219.0672, 177.0204	C_26_H_28_O_7_	6,2′-diprenylquercetin-3-methyl ether (42)	[M-H]^−^	-
50^a^	16.144	383.1126	383.1136	−2.61	368.0288, 312.0267, 284.0276	C_21_H_20_O_7_	5′-prenylquercetin-3-methyl ether (4)	[M-H]^−^	-
51^b*^	16.158	411.1087	411.1085	0.49	379.0825, 351.0835, 219.0652	C_22_H_20_O_8_	podophyllotoxne (43)	[M-H]^−^	-
52^a^	16.501	467.1704	467.1711	−1.50	449.1621, 393.0994, 247.0621, 175.4930	C_26_H_28_O_8_	dysosmaflavone E (4)	[M-H]^−^	√
53^a^	16.596	353.1024	353.1031	−1.98	298.0455, 253.0451, 225.0525, 219.0689, 163.0048, 136.0178	C_20_H_18_O_6_	8-prenylkaempferol (4)	[M-H]^−^	√
54^a^	16.700	383.1147	383.1136	2.87	339.1289, 324.1010, 173.0610	C_21_H_20_O_7_	dimethoxyflavonol (4)	[M-H]^−^	√
55^a^	16.750	437.1595	437.1606	−2.52	368.0913, 247.0609, 219.0668, 176.0129, 133.0663	C_25_H_26_O_7_	broussonol E (4)	[M-H]^−^	-
56^a^	16.838	383.1145	383.1136	2.35	325.0369, 308.0334, 254.0474, 152.0116, 151.1032	C_21_H_20_O_7_	sinoflavonoid F (4)	[M-H]^−^	√
57^a^	16.922	367.0843	367.0823	5.45	312.0269, 296.0304, 283.0258, 242.0209, 217.0521, 173.0590	C_20_H_16_O_7_	7,8-(2″,2″-dimethyl pyrane)-3,5,3′,4′-tetrahydroxy flavone or isomer (4)	[M-H]^−^	√
58^a^	17.054	383.1120	383.1136	−4.18	353.0888, 351.0892, 178.9993, 151.0038	C_21_H_20_O_7_	4′,5′-(2″,2″-dimethyl-3″,4″-dihydropyran)-5,7,3′-trihydroxy-3-methoxy flavone (4)	[M-H]^−^	√
59^a^	17.237	383.1133	383.1136	−0.78	315.0455, 314.0407, 300.0256, 299.0202	C_21_H_20_O_7_	5,7,3′-trihydroxy-3-methoxy flavone-4′-prenyl ether or isomer (4)	[M-H]^−^	√
60^a^	17.416	451.1756	451.1762	−1.33	247.0623, 248.0685, 219.0675, 177.0204, 151.0770	C_26_H_28_O_7_	8,2′-diprenylquercetin-3-methyl ether (4)	[M-H]^−^	-
61^a^	17.465	367.0837	367.0823	3.81	339.0904, 312.0313, 217.0516, 173.0612	C_20_H_16_O_7_	7,8-(2″,2″-dimethyl pyrane)-3,5,3′,4′-tetrahydroxy flavone or isomer (4)	[M-H]^−^	-
62^a^	17.482	451.1766	451.1762	0.89	393.0994, 247.0629, 219.0672	C_26_H_28_O_7_	6,6′-diprenylquercetin-3-methyl ether (4)	[M-H]^−^	-
63^a^	17.837	351.0862	351.0874	−3.42	323.0950, 295.0990, 217.0524, 189.0544, 173.0609, 161.0616	C_20_H_16_O_6_	citrusinol (4)	[M-H]^−^	-
64^a^	17.847	367.1171	367.1187	−4.36	352.0973, 309.0425, 297.0380, 267.0333	C_21_H_20_O_6_	2′-prenylkaempferol-3-methyl ether (4)	[M-H]^−^	√
65^b*^	18.048	415.1384	415.1387	−0.72	247.0671, 189.0633, 229.0257	C_22_H_22_O_8_	podophyllotoxin (4)	[M + H]+	-
66^a^	18.078	367.1185	367.1187	−0.54	352.0963, 323.0918, 309.0416, 297.0434, 295.0979, 239.0347	C_21_H_20_O_6_	6-prenylkaempferol-3-methyl ether (44)	[M-H]^−^	-
67^a^	18.211	367.0815	367.0823	−2.18	352.0953, 309.0353, 297.0333, 245.0475, 173.0606	C_20_H_16_O_7_	7,8-(2″,2″-dimethyl pyrane)-3,5,3′,4′-tetrahydroxy flavone or isomer (4)	[M-H]^−^	-
68^b^	18.275	397.1273	397.1293	−5.04	313.0726, 188.0849, 179.0039, 165.0177, 151.0032	C_22_H_22_O_7_	desoxypodophyllotoxin (23)	[M-H]^−^	-
69^a^	18.605	367.1175	367.1187	−3.27	352.0978, 309.0414, 253.0514	C_21_H_20_O_6_	3′-prenylkaempferol-3-methyl ether (9, 10)	[M-H]^−^	-
70^b^	18.707	411.1072	411.1085	−3.16	379.0803, 327.0136, 180.0059, 152.0122	C_22_H_20_O_8_	podophyllotoxne isomer (4)	[M-H]^−^	√
71^a^	18.868	451.1763	451.1762	0.22	247.0620, 219.0674, 177.0202, 151.0768, 133.0663	C_26_H_28_O_7_	8,6′-diprenylquercetin-3-methyl ether (4)	[M-H]^−^	-
72^a^	18.880	367.1183	367.1187	−1.09	309.0398, 297.0402, 253.0500, 225.0581	C_21_H_20_O_6_	8-prenylkaempferol-3-methyl ether (45)	[M-H]^−^	-
73^a^	19.458	451.1759	451.1762	−0.66	368.0840, 247.0626, 219.0672, 177.0205, 163.0026	C_26_H_28_O_7_	6,5′-diprenylquercetin-3-methyl ether (4)	[M-H]^−^	-
74^a^	19.732	435.1432	435.1449	−3.91	417.1320, 287.1008, 219.0671	C_25_H_24_O_7_	8,2′-diprenylkaempferol-3-methyl ether (46)	[M-H]^−^	-
75^b^	19.771	379.0844	379.0823	5.54	364.0566, 335.0573, 319.1958, 199.0734	C_21_H_16_O_7_	diphyllin (23)	[M-H]^−^	-
76^a^	19.823	435.1446	435.1449	−0.69	349.0341, 326.0420, 325.0352	C_25_H_24_O_7_	7,8-(2″,2″-dimethyl pyrane)-6′-prenyl-3,5,3′,4′-tetrahydroxy flavone (4)	[M-H]^−^	√
77^b^	19.918	399.1444	399.1438	1.50	231.0681, 203.0779, 187.0812	C_22_H_22_O_7_	desoxypodophyllotoxin (4)	[M + H]^+^	-
78^a^	20.006	449.1600	449.1606	−1.34	218.0583, 217.0517, 203.0355	C_26_H_26_O_7_	7,8-(2″,2″-dimethyl pyrane)-2′-prenyl-5,3′,4′-trihydroxy-3-methoxy flavone (9, 10)	[M-H]^−^	-
79^a^	20.170	451.1753	451.1762	−1.99	436.1535, 393.0982, 367.0831, 325.0345	C_26_H_28_O_7_	8,5′-diprenylquercetin-3-methyl ether (4)	[M-H]^−^	-
80^a^	20.235	449.1610	449.1606	0.89	218.0587, 217.0515, 173.0613, 131.0504	C_26_H_26_O_7_	8-prenyl-2′,3′-(2,2-dimethyl pyrane)-5,7,4′-trihydroxy-3-methoxy flavone (9, 10)	[M-H]^−^	-
81^b^	20.398	385.1283	385.1283	0.00	231.0621, 173.0638, 187.0511	C_21_H_20_O_7_	4′-demethyldeox Ypodophyllotoxin (4)	[M + H]^+^	-
82^a^	21.578	449.1603	449.1606	−0.67	375.0838, 332.0329, 321.0394, 320.0328, 247.0628, 219.0674	C_26_H_26_O_7_	6-prenyl-3′,4′-(2,2-dimethyl pyrane)-5,7,5′-trihydroxy-3-methoxy flavone (4)	[M-H]^−^	-
83^a^	21.587	367.0820	367.0823	−0.82	339.0887, 312.0284, 311.0930, 284.0355, 283.0232, 217.0525, 173.0624	C_20_H_16_O_7_	7,8-(2″,2″-dimethyl pyrane)-3,5,3′,4′-tetrahydroxy flavone or isomer (4)	[M-H]^−^	-
84^a^	21.761	449.1607	449.1606	0.22	375.0890, 320.0316, 219.0673	C_26_H_26_O_7_	8-prenyl-3′,4′-(2,2-dimethyl pyrane)-5,7,5′-trihydroxy-3-methoxy flavone (47)	[M-H]^−^	-
85^a^	23.211	447.1434	447.1449	−3.35	217.0491, 216.0444, 215.0358, 188.0499, 187.0381	C_26_H_24_O_7_	7,8-(2″-isopropenyl-furano)-2′-prenyl-5,3′,4′-trihydroxy-3-methoxy flavone (9, 10)	[M-H]^−^	-

a Represents flavonoids.

b Represents lignans.

“*“*” indicates that the changed compound has been compared with the control product.

Since FSH mainly contain flavonoids and lignin compounds, the cleavage patterns of certain compounds in the above two classes were investigated.

#### 3.2.1 Partial cleavage patterns of flavonoids

Peaks 5, 12 and 16: the t_R_ of peak 5 was 6.838 min, while those of peaks 12 and 16 were 7.883 min and 9.223 min, respectively. In addition, the quasimolecular ion peaks in the negative ion mode were m/z 477.1053[M-H]^−^, m/z 477.1024[M-H]^−^ and m/z 477.1045[M-H]^−^, respectively. The molecular formula was C_22_H_22_O_12_, and the relative molecular mass was 478.1111, indicating that the three were isomers. In the secondary MS of the negative ions, the fragment ion peaks m/z 315.0523, m/z 315.0520, and m/z 315.0495 were observed, all of which were the glycosidic fragment ions obtained by removing one molecule of glucose. The ion fragment peaks m/z 300.0276, m/z 300.0277 and m/z 300.0226 obtained by removing one molecule of CH_3_ on the top of the glycoside fragment ion indicated the presence of methoxy substitution in the molecular backbone of the glycoside. Besides, a series of ion fragments m/z 271.0251 and m/z 255.0227 were found in the secondary mass spectra of the negative ions as the typical mass spectral cleavage features of the flavonoid glycosidic skeleton. The three fragment ions were roughly the same, with relative differences in their respective ion abundances. The above fragmentation information of the flavonoid glycoside element basically agreed with the mass spectral cleavage pattern of quercetin. According to the relevant reference (19), peak 5 was presumed to be quercetin-3-methyl ether-3′/4’-O-glucoside, peak 12 was quercetin-3-methyl ether-3′/4’-O-glucoside, and peak 16 was quercetin-3-methyl ether-7-O-glucoside.

Peak 6: The t_R_ of peak 6 was 6.981 min, and the quasimolecular ion peak in the negative ion mode was m/z 609.1458[M-H]^−^, which was deduced to have a molecular formula of C_27_H_30_O_16_ and a relative molecular mass of 610.1534. In the secondary MS of the negative ions, the ion fragment m/z 463.0088 was the fragmented ion formed by the detachment of rhamnose from the compound, while the ion fragments m/z 301.0326 and m/z 300.0278, as well as m/z 271.0282 and m/z 255.0291 were the fragmentation of glycosides obtained from the continued removal of glucose by this ion. The above fragment ion information was basically consistent with the MS cleavage pattern of rutin, meanwhile, according to the related reference (20), peak 6 was presumed to be rutin.

**Figure 3:**
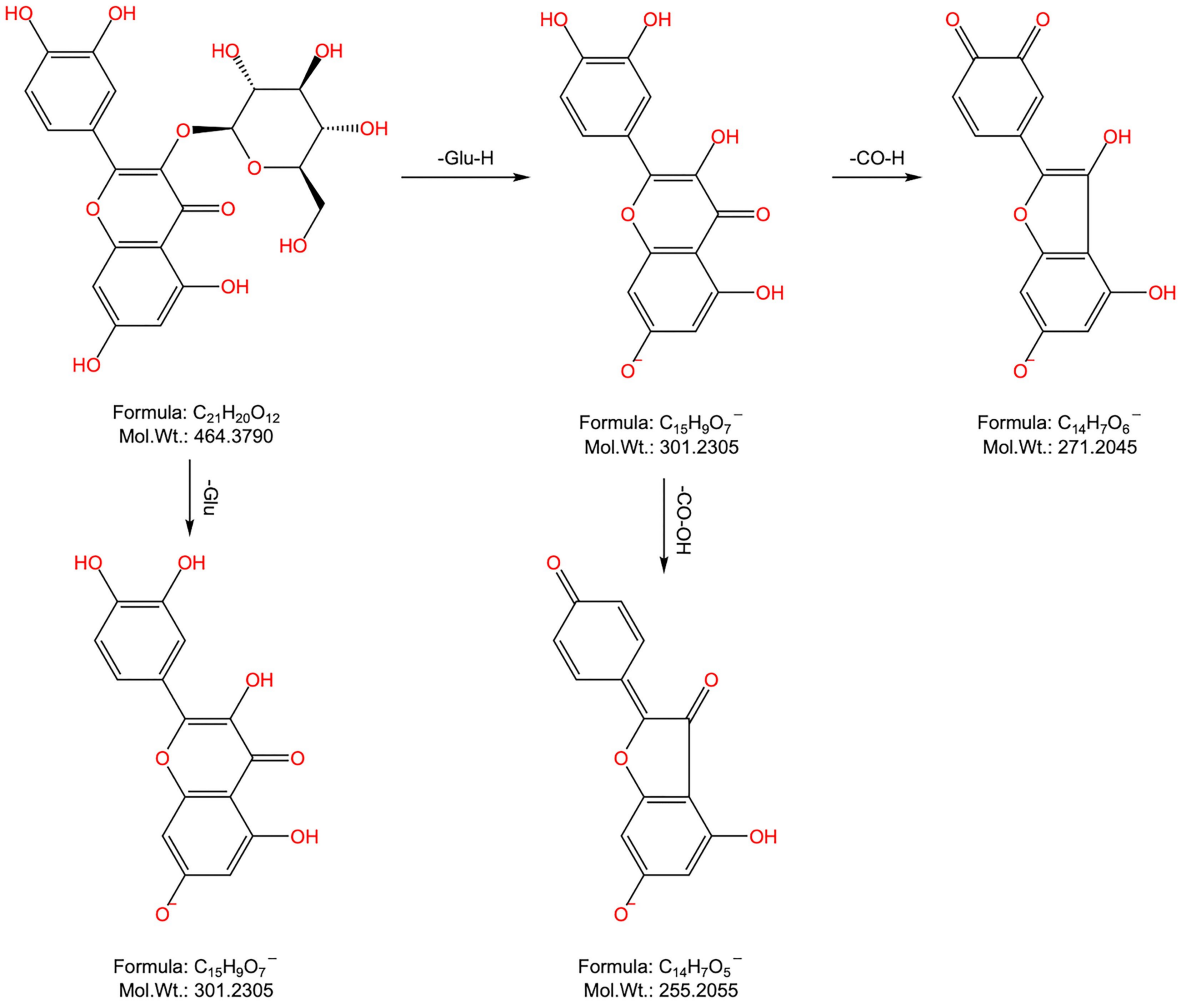
Chemical structure and major cleavage fragments of quercetin-3-O-glucoside.

Peak 8: Its t_R_ was 7.363 min, and the quasimolecular ion peak in the negative ion mode was m/z 463.0872[M-H]^−^, which was inferred to have a molecular formula of C_21_H_20_O_12_ and a relative molecular mass of 464.0955. In the secondary MS of the negative ions, the ion fragment m/z 301.0326 was obtained through stripping off a molecule of C_6_H_10_O_5_, and the compound was suspected to be flavonoid glucoside. At the same time, ion fragments m/z 271.0282 and m/z 255.0291 were also found in the secondary spectrum, consistent with the mass spectral cleavage pattern of quercetin (Figure 3). Combined with the ion fragments of the control product and the relevant references (4, 7), it was hypothesized that peak 8 was attributed to quercetin-3-O-glucoside.

Peaks 18 and 57: Their t_R_ values were 9.917 min and 16.922 min, respectively, and the quasimolecular ion peaks in the negative ion mode were m/z 367.0810[M-H]^−^ and m/z 367.0843[M-H]^−^, respectively, which inferred that both of them had a molecular formula of C_20_H_16_O_7_ and a relative molecular mass of 368.0896, suggesting that the two were isomers. In the negative ion secondary mass spectra, the ion fragments m/z 352.0520, 293.0688, 266.0580, and 193.0146 for peak 8 were observed. Meanwhile, the ion fragments m/z 312.0269, 296.0304, 283.0258, 242.0209, 217.0521, and 173.0590 of peak 31 were seen. Based on the secondary MS fragment ion data of the above two compounds and combined with the related literature reports (4), it was hypothesized that peak 18 was 2′,3′-(2″,2″-dimethyl pyrane)-3,7,5,4′-tetrahydroxy flavone or isomer, whereas peak 57 was 7,8-(2″,2″-dimethyl pyrane)-3,5,3′,4′-tetrahydroxy flavone or isomer.

Peak 25: The t_R_ was 10.928 min, and the quasimolecular ion peak in the negative ion mode was m/z 315.0502[M-H]^−^, which was inferred to have a molecular formula of C_16_H_12_O_7_ and a relative molecular mass of 316.0583. In the negative ion secondary MS, fragment ions were found, such as m/z 271.0288, 255.0311, and 227.0365, which represented the typical fragments of the quercetin backbone structure. Also, based on the m/z 300.0293 plasma fragment, the control fragment ions and the related literature reports (21), it was hypothesized that peak 25 was quercetin-3-methyl ether.

Peak 30: Its t_R_ was 12.393 min, and the quasimolecular ion peak in the negative ion mode was m/z 285.0407[M-H]^−^, which was inferred to have a molecular formula of C_15_H_10_O_6_ and a relative molecular mass of 286.0477. In the negative ion secondary MS, the fragment ion m/z 255.0256 was formed from the parent ion by removing the CO from the C-ring, and the fragment ion m/z 239.0342 was obtained by continuing to remove O after the removal of CO from the parent ion, while the fragment ion m/z 211.0385 was formed through removing CO_2_ from the parent ion and then removing CO again. In addition, the fragment ion m/z 133.0299 formed after the cleavage of RDA was also found. Combined with the relevant literature reports (4), it was hypothesized that peak 30 was associated with the compound kaempferol.

**Figure 4:**
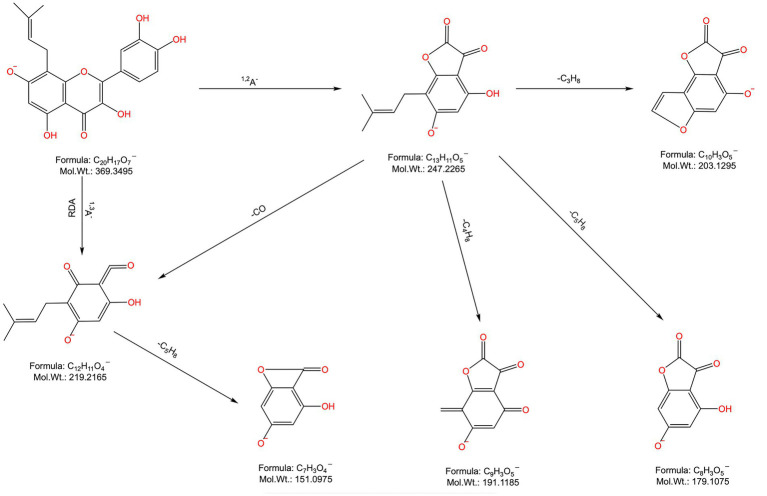
Chemical structure and major cleavage fragments of 8-prenylquercetin.

Peaks 37 and 39: Their t_R_ values were 14.6 min and 14.875 min, respectively, and the quasimolecular ion peaks in the negative ion mode were 369.0967 [M-H]^−^ and 369.0949 [M-H]^−^, separately, which were predicted to have a molecular formula of C_20_H_18_O_7_ and a relative molecular mass of 370.1053, indicating that the two were isomers. In the negative ion secondary MS, fragment ions m/z 351.0850, 323.0866, 299.0530, and 255.0265 were observed in peak 19, which, combined with the relevant literature report (4), were presumed to be uralenol in peak 37. Fragment ion m/z 219.0688, which could be found in peak 39, was typical of the flavonoid glycoside element RDA. The fragment ion m/z 300.0257 was obtained from the parent ion by removing the isopentenyl group. Additionally, the characteristic fragment ions m/z 313.0386, 247.0572, 219.0688, 202.9968, 191.0014, 179.0004, and 151.0779 were also found (Figure 4). Combined with the relevant literature report (4), peak 39 was hypothesized to be 8-prenylquercetin.

Peaks 40, 47 and 48: Their t_R_ were 15.084 min, 15.961 min and 16.025 min, respectively. The quasimolecular ion peaks in the negative ion mode were m/z 383.1150[M-H]^−^, m/z 383.1156[M-H]^−^, m/z 383.1152[M-H]^−^, and m/z 383.1152[M-H]^−^. It was deduced that the three were isomers with a molecular formula of C_21_H_20_O_7_, and a relative molecular mass of 384.1209. In the secondary sub-spectrum of the negative ion of peak 40, the fragment ion m/z 368 was obtained from the parent ion stripped off CH_3_, the fragment ion m/z 339 was derived from the parent ion by removing CO_2_, the fragment ion m/z 325 was acquired from the parent ion by eliminating C_4_H_10_, and the fragment ion m/z 175 was obtained from the parent ion. Moreover, the fragment ions m/z 369.0947, 326.0401, 314.0385, and 176.0136 were also found, which were presumed to be 6-isopentenylquercetin-3-methyl ether by combining with the related literature report (4). In the secondary MS of the negative ion of peak 47, the fragment ion m/z 368.0913 was obtained from the parent ion by removing one molecule of methyl ether, and the fragment ion m/z 325 was acquired from the parent ion by removing C_4_H_10_, whereas the fragment ion m/z 175 was derived from the parent ion by removing C_13_H_20_O_2_. The fragment ion m/z 368.0913 was produced from the parent ion by removing one molecule of methyl radical, and the fragment ion m/z 339.0899 was generated from the parent ion by removing one molecule of CO_2_. Additionally, the fragment ions m/z 369.0950, 323.0925, 284.0276, and 283.0244 were also detected, which, combined with the relevant literature report (4), were presumed to be 6′ -isopentenylquercetin-3-methyl ether. In the secondary MS of the negative ion of peak 48, the fragment ion m/z 368.0917 was formed from the parent ion by removing the methyl group, while the fragment ion m/z 325.0364 was obtained from the parent ion by eliminating the C_4_H_10_ group, and the fragment ion m/z 175.0053 was acquired from the parent ion by removing the C_13_H_20_O_2_ group. By comparison with other fragment ions and combination with literature report (4), peak 48 was presumed to be 8-isopentenylquercetin-3-methyl ether.

Peaks 43 and 46: Their t_R_ were 15.67 min and 15.944 min, respectively, and the quasimolecular ion peaks in the negative ion mode were m/z 299.0555[M-H]^−^ and m/z 299.0562[M-H]^−^, separately. It was deduced that the two were isomers with a molecular formula C_16_H_12_O_6_ and a relative molecular mass of 300.0634. In the two negative ion secondary mass spectra, the fragment ions m/z 284.0350 and m/z 284.0365 were found to be obtained by removing CH_3_ from the corresponding parent ions. Meanwhile, the same fragment ions m/z 227.0363 and m/z 227.0364, as well as the related ion fragments m/z 255.0308, 167.0518, 164.0098, 151.0518 164.0098, and 151.0046 were also found, which, combined with relevant literature reports (22, 23), speculated that peak 43 was kaempferol-3-methyl ether and peak 46 was kaempferol-4′-methyl ether.

Peak 64: The t_R_ was 17.847 min, and the quasimolecular ion peak in the negative ion mode was m/z 367.1171[M-H]^−^, and peak 64 was inferred to have a molecular formula of C_21_H_20_O_6_ and a relative molecular mass of 368.1260. In the negative ion secondary MS, the fragment ion m/z 352.0973 was obtained from the parent ion by removing CH_3_, and the fragment ion m/z 309.0425 was generated by the continuous removal of C_3_H_7_ from the parent ion, while the fragment ion m/z 297.0380 was produced from the parent ion by continuing to remove C_4_H_7_ after the removal of CH_3_. Additionally, the fragment ion m/z 309.0425 was obtained when the parent ion continued to remove C_3_H_7_, and the fragment ion m/z 297.0380 was acquired when the parent ion further removed C_4_H_7_ after removing CH_3_. Combined with the above ion fragmentation information and related literature report (4), it was hypothesized that peak 64 was 2′-prenylkaempferol-3-methyl ether.

#### 3.2.2 Partial cleavage patterns of lignin analogs

Peak 70: The t_R_ was 18.707 min, and the quasimolecular ion peak in the negative ion mode was m/z 411.1072[M-H]^−^, which was inferred to have a molecular formula of C_22_H_20_O_8_ and a relative molecular mass of 412.1158. In the negative ion secondary MS, the characteristic fragment ion m/z 379.0803 and fragment ion m/z 297.0345 formed by the cleavage rearrangement of the bynyl group were also detected in addition to fragment ions m/z 327.0136, 180.0059, and 152.0122. Combined with the related literature report (4), it was hypothesized that peak 70 was the podophyllotoxne isomer.

### 3.3 Analysis of the *in vivo* absorbed and transformed chemical components of FSH

The mass spectra of rat drug-containing plasma samples and blank plasma samples were compared. Combined with the MS analysis patterns of the chemical components of FSH, a total of 35 prototype components and 13 *in vivo* transformed products were identified in the rat plasma based on MS data. The mass spectra of blank plasma and drug-containing plasma samples are shown in Figures 2C–L. More details of the *in vivo* absorbed components are displayed in Table 1, and the *in vivo* transformed products are presented in Table 2.

**Table 2:** The *in vivo* absorbed and transformed chemical components of FSH.

No.	t_R_/min	Measured value (m/z)	Theoretical value (m/z)	ppm	Molecular formula	Chemical compound	Ionization mode
M1	2.446	409.0914	409.0929	−3.67	C_22_H_18_O_8_	Dehydrogenation products of podophyllotoxne	[M-H]^−^
M2	3.094	577.1538	577.1563	−4.33	C_27_H_30_O_14_	Oxygen-depleted product of kaempferol-3-O-rutinoside	[M-H]^−^
M3	3.310	607.1650	607.1668	−2.96	C_28_H_32_O_15_	Kaempferol-3-O-rutinoside methylation product	[M-H]^−^
M4	3.409	671.1980	671.1981	−0.15	C_32_H_34_O_13_	7,8-(2″,2″-Dimethylpyrane)-2′-prenyl-5,3′,4′-trihydroxy-3-methoxy flavone glucuronidation product	[M + COOH]^−^
M5	3.487	575.1382	575.1406	−4.17	C_27_H_28_O_14_	α-peltatin or isomer glucuronidation product	[M-H]^−^
M6	3.891	461.0718	461.0725	−1.52	C_21_H_18_O_12_	Kaempferol glucuronide product	[M-H]^−^
M7	4.569	491.0809	491.0831	−4.48	C_22_H_20_O_13_	Quercetin-3-methyl ether glucuronide product	[M-H]^−^
M8	4.660	559.1443	559.1457	−2.50	C_27_H_28_O_13_	4′-demethyldeoxypodophyllotoxin glucuronide product	[M-H]^−^
M9	5.216	545.1288	545.1301	−2.38	C_26_H_26_O_13_	8-Prenylquercetin glucuronide product	[M-H]^−^
M10	6.879	627.2069	627.2083	−2.23	C_32_H_36_O_13_	8, 6’-Diprenylquercetin-3-methyl ether glucuronide product	[M-H]^−^
M11	7.751	447.0918	447.0933	−3.36	C_21_H_20_O_11_	Quercetin-3-methyl ether-7-O-glucoside hydroxymethylene loss products	[M-H]^−^
M12	28.118	395.1498	395.1500	−0.51	C_23_H_24_O_6_	2’-Prenylkaempferol-3-methyl ether dimethylated product	[M-H]^−^
M13	28.799	421.1651	421.1657	−1.42	C_25_H_26_O_6_	Broussonol E Eoxygen loss products	[M-H]^−^

**Figure 5:**
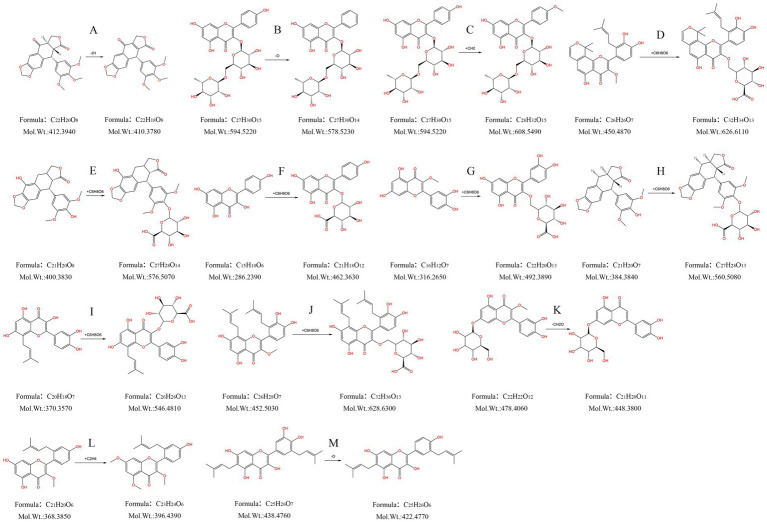
Metabolism pattern of metabolites in FSH. **(A)** Dehydrogenation products of podophyllotoxne. **(B)** Oxygen-depleted product of kaempferol-3-O-rutinoside. **(C)** Kaempferol-3-O-rutinoside methylation product. **(D)** 7,8-(2″,2″-Dimethylpyrane)-2’-prenyl-5,3’,4’-trihydroxy-3- methoxy flavone glucuronidation product. **(E)** α-peltatin or isomer glucuronidation product. **(F)** Kaempferol glucuronide product. **(G)** Quercetin-3-methyl ether glucuronide product. **(H)** 4’-demethyldeoxypodophyllotoxin glucuronide product. **(I)** 8-Prenylquercetin glucuronide product. **(J)** 8, 6’-Diprenylquercetin-3- methyl ether glucuronide product. **(K)** Quercetin-3-methyl ether-7-O-glucoside hydroxymethylene loss products. **(L)** 2’-Prenylkaempferol-3- methyl ether dimethylated product. **(M)** Broussonol E Eoxygen loss products.

Compound M1: The t_R_ was 2.446 min, the quasimolecular ion peak in the negative ion mode was m/z 409.0914, and its molecular formula was inferred to be C_22_H_18_O_8_, which differed from the quasimolecular ion peak in the negative ion mode of compound 51 of m/z 411.1087 by 2 Da. It was suggested that compound 51 was dehydrogenated from H_2_ in rats, therefore, compound M1 was inferred to be the dehydrogenated product of compound 51, as shown in Figure 5A.

Compound M2: The t_R_ was 3.094 min, and the quasimolecular ion peak in the negative ion mode was m/z 577.1538, which was inferred to have a molecular formula of C_27_H_30_O_14_. In the meantime, the quasimolecular ion peak in the negative ion mode of compound 10 was m/z 593.1507, with a difference of 16 Da compared with compound M2. Consequently, compound M2 was speculated to be the dehydrogenated product of compound 10 in rats, as observed from Figure 5B.

Compound M3: The t_R_ was 3.310 min, and the quasimolecular ion peak in the negative ion mode was m/z 607.1650. Its molecular formula was deduced to be C_28_H_32_O_15_, which was 14 Da different from the quasimolecular ion peak in the negative ion mode of compound 10, m/z 593.1507. It was speculated that compound 10 increased CH_3_ in rats, therefore compound M3 was assumed to be the methylated product of compound 10, as shown in Figure 5C.

Compound M4: Its t_R_ was 3.409 min, its quasimolecular ion peak in the negative ion mode was m/z 671.1980, and it was inferred to have a molecular formula of C_32_H_34_O_13_, which differed by 221 Da from the quasimolecular ion peak of compound 78 in the negative ion mode (m/z 449.1600). Consequently, it was presumed that compound M4 was the methylated product of compound 10, namely, the dimethylpyrane-2′-prenyl-5,3′,4′-trihydroxy-3-methoxy flavone glucuronidation product (Figure 5D).

Compound M5: The t_R_ was 3.487 min, the quasimolecular ion peak in the negative ion mode was m/z 575.1382, and it was inferred with a molecular formula of C_27_H_28_O_14_. Besides, the quasimolecular ion peak in the negative ion mode of compound 4′-demethylprenyl glucuronide had was m/z 399.1085, which differed from compound M5 by 176 Da. In this regard, it was inferred that compound M5 was the 4′-demethyldauricin glucuronidated product, as observed from Figure 5E.

Compound M6: The t_R_ was 3.891 min, the quasimolecular ion peak in the negative ion mode was m/z 461.0718, and its molecular formula was inferred to be C_21_H_18_O_12_, which was 176 Da different from the quasimolecular ion peak in the negative ion mode of compound 30 (m/z 285.0407). As a result, it was presumed that compound M6 was a kaempferol glucuronidated product (Figure 5F).

Compound M7: The t_R_ was 4.569 min, and the quasimolecular ion peak in the negative ion mode was m/z 491.0809, which suggested that the molecular formula of compound M7 was C_22_H_20_O_13_, with a difference of 176 Da compared with the quasimolecular ion peak in the negative ion mode of compound 25 (m/z 315.0502). Consequently, compound M7 was presumed to be the quercetin-3-methyl ether glucuronidated product (Figure 5G).

Compound M8: The t_R_ was 4.660 min, the quasimolecular ion peak in the negative ion mode was m/z 559.1443, and its molecular formula was inferred to be C_27_H_28_O_13_, which differed from the quasimolecular ion peak in the negative ion mode of compound 4′-demethyldeoxypodophyllotoxin by 176 Da. Therefore, compound M8 was presumed to be the 4′-demethyldeoxypodophyllotoxin glucuronide product, as observed from Figure 5H.

Compound M9: The t_R_ was 5.216 min, and the quasimolecular ion peak in the negative ion mode was m/z 545.1288, inferring that compound M9 had a molecular formula of C_26_H_26_O_13_. Additionally, there was a difference of 176 Da compared with the quasimolecular ion peak in the negative ion mode of compound 39 (m/z 369.0980). So it was speculated that compound M9 was the 8-prenyl quercetin glucuronidated product (Figure 5I).

Compound M10: The t_R_ was 6.879 min, and the quasimolecular ion peak in the negative ion mode was m/z 627.2069, which was deduced to have a molecular formula C_32_H_36_O_13_. There was a difference of 176 Da compared with the quasimolecular ion peak in the negative ion mode of compound 60 (m/z 451.1762). In this regard, it was hypothesized that compound M10 was the 8,6′-diprenylquercetin-3-methyl ether glucuronidated product (Figure 5J).

Compound M11: The t_R_ was 7.751 min, and the quasimolecular ion peak in the negative ion mode was m/z 447.0918, which was inferred to have a molecular formula of C_21_H_20_O_11_. A difference of 30 Da was found compared with the quasimolecular ion peak of compound 16 in the negative ion mode (m/z 477.1038). Therefore, compound M11 was assumed to be the quercetin-3-methyl ether-7-O-glucoside loss product of hydroxymethylene, as observed from Figure 5K.

Compound M12: The t_R_ was 28.118 min, and the quasimolecular ion peak in the negative ion mode was m/z 395.1498. Its molecular formula was inferred to be C_23_H_24_O_6_, which was 28 Da different from the quasimolecular ion peak in the negative ion mode of compound 64 (m/z 367.1171). Thus, compound M12 was inferred to be the 2′-prenylkaempferol-3-methyl ether dimethylated product, as shown in Figure 5L.

Compound M13: Its t_R_ was 28.799 min, and its quasimolecular ion peak in the negative ion mode was m/z 421.1651, which was inferred to have a molecular formula of C_25_H_26_O_6_. Meanwhile, a difference of 16 Da was detected compared with the quasimolecular ion peak in the negative ion mode of compound 55 (m/z 437.1595). Consequently, compound M13 was inferred to be the oxygen-loss product of broussonol E (Figure 5M).

### 3.4 Network pharmacology of the anti-tumor effect of FSH

#### 3.4.1 Acquisition of the *in vivo* transformed components and targets of FSH

Based on the molecular structures of the 35 prototypical absorbed components and 13 prototypical components of *in vivo* transformed metabolites identified in FSH, their potential targets were predicted based on their chemical structural formulas by swiss target prediction (see text footnote 2), and finally after de-weighting, a total of 319 drug-related targets for 25 components were obtained.

#### 3.4.2 Acquisition of the tumor disease-related targets

Using “tumor” as the keyword, the Gene Cards database and OMIM database were searched, respectively, which obtained 1,567 disease-related targets after taking the concatenated set.

#### 3.4.3 Acquisition of the intersected targets between FSH and the tumor disease

**Figure 6:**
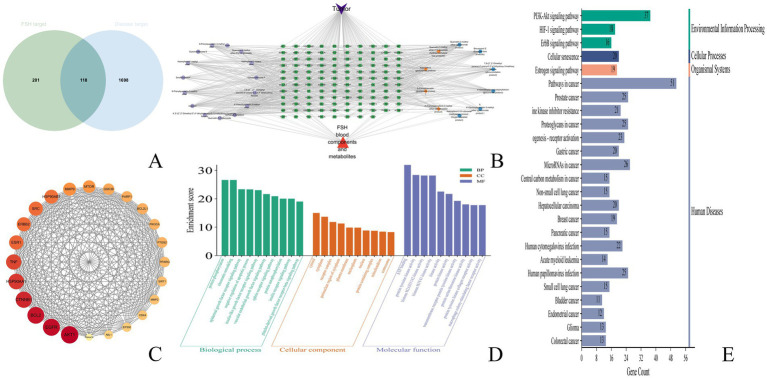
Anti-tumor network pharmacological results of FSH. **(A)** veen plot. **(B)** drug-component-target plot. **(C)** Protein–protein interaction (PPI) network plot. **(D)** GO pathway analysis plot. **(E)** KEGG pathway analysis plot.

The tumor-related targets were mapped with the transformed components of FSH by adopting the microbiology data analysis platform, and 118 intersected targets were obtained, as shown in Figure 6A.

#### 3.4.4 Construction of the drug-component-target-disease network

The transformed components and drug-disease intersected targets of FSH were imported into Cytoscape 3.9.1 software, and the “drug-component-target” map was plotted in Figure 6B. In this visualization, green nodes represent drug-disease intersected targets, cyan nodes denote the prototype component of the transformed component of FSH *in vivo*, orange nodes indicate components where both the prototype component and the metabolite were observed, and blue nodes signify components where only the metabolite was detected. Subsequently, utilizing the predicted core targets, the associated chemical components were quantified within Cytoscape 3.9.1 software. The top ten core components were identified based on a Degree value threshold of ≥40, as detailed in Table 3.

**Table 3:** Core components of FSH for tumor therapy.

No.	Degree	Core component
1	53	6’-Prenylquercetin-3-methyl ether
2	49	8-Prenylquercetin (glucuronide product)
3	49	Quercetin-3-methyl ether (glucuronide product)
4	48	Uralenol
5	46	Kaempferol-4′-methyl ether
6	45	Kaempferol (glucuronide product)
7	44	6-Prenylquercetin-3-methyl ether
8	44	Kaempferol-3-methyl ether
9	43	4’-Demethyldeoxypodophyllotoxin (glucuronide product)
10	43	Broussonol E (Eoxygen loss products)

#### 3.4.5 Protein–protein interaction network analysis

The 113 intersected targets of FSH and tumor disease were imported into the String database to construct the PPI network. After removing the free nodes, the network topology was analyzed using Cytoscape 3.9.1 software, and then the topological parameters of the network nodes were analyzed by the Network Analyzer plug-in. Using the three parameters of BC, CC and Degree as the indicators, the medians of these three indicators were calculated. When the target indicator was larger than the corresponding median, it was predicted as a core target. Finally, the core targets were ranked in accordance with the Degree value. There were 24 nodes and 255 edges in the network, as observed from Figure 6C and Table 4.

**Table 4:** Core targets of FSH for tumor therapy.

No.	Degree	Core targets
1	95	AKT1
2	93	EGFR
3	92	BCL2
4	89	CTNNB1
5	86	HSP90AA1
6	84	TNF
7	79	ESR1
8	78	ERBB2
9	78	SRC
10	77	HSP90AB1

#### 3.4.6 GO and KEGG enrichment analyses

Upon GO functional annotation, totally 612 biological processes (BP) were analyzed, including protein phosphorylation, chromatin remodeling, epidermal growth factor receptor signaling pathway, and negative regulation of apoptotic process. Meanwhile, 100 cellular components (CC) were obtained, including cytosol, cytoplasm, receptor complex, perinuclear region of cytoplasm, and plasma membrane. There were also 179 molecular functions (MF) being acquired, including ATP binding, protein tyrosine kinase activity, histone H2AXY142 kinase activity, and histone H3Y41 kinase activity. The data were analyzed by the Microbiology Data Analysis Platform (MDA). The bar chart of GO functional enrichment analysis was produced by the Microbiotics data analysis platform (Figure 6D).

As suggested by the KEGG pathway enrichment analysis results, there were 146 pathways enriched by the tumor treatment with FSH. The top 25 KEGG pathways were input into the MDA platform, and the bubble diagram showing the KEGG pathway enrichment analysis results was produced (Figure 6E). Obviously, the main signaling pathways engaged in the targets of FSH for tumor treatment were Pathways in cancer, Prostate cancer, PI3K-Akt signaling pathway, EGFR tyrosine kinase inhibitor resistance, and Proteoglycans in cancer. The results of the top 25 KEGG pathways enriched were summarized with their associated chemical compositions, and the results are presented in Supplementary Table S1.

After combining the top 25 KEGG results with PPI data, it was observed that AKT1 was present in 23 pathways, with the exceptions of “MicroRNAs in cancer” and “Bladder cancer.” EGFR was identified in 22 pathways, excluding “Cellular senescence,” “Acute myeloid leukemia,” and “Small cell lung cancer.” BCL2 appeared in 11 pathways, including “Pathways in Cancer” and “Prostate Cancer,” among others. CTNNB1 was involved in 10 pathways, such as “Prostate Cancer” and “Proteoglycans in Cancer.” HSP90AA1 was found in “Pathways in Cancer,” “Prostate Cancer,” and five additional pathways. TNF was associated with 3 pathways: “Proteoglycans in Cancer,” “Human Cytomegalovirus infection,” and “Human Papillomavirus infection.” ESR1 was included in 5 pathways, notably “Pathways in Cancer” and “Proteoglycans in Cancer.” ERBB2 was involved in 15 pathways, including “Pathways in Cancer” and “Prostate Cancer.” SRC was present in “EGFR tyrosine kinase inhibitor resistance,” “Proteoglycans in Cancer,” and seven other pathways. Lastly, HSP90AB1 was included in five pathways, such as “Pathways in Cancer” and “Prostate Cancer”.

### 3.5 Anti-tumor efficacy of chemical components in FSH

The inhibition results of Uralenol and Kaempferol on different tumor cells, as detected by the CCK-8 method, are shown in Tables 5–8. The results showed that Uralenol and Kaempferol exert good inhibitory effects on human hepatoma cell line HepG2, human non-small cell lung cancer cell line A549, human colon cancer cell line SW620, and mouse breast cancer cell line 4T1, which verified the reliability of the network pharmacology results.

**Table 5:** The inhibitory effect of different monomers on HepG2 cells.

Ingredient name	Concentration (μmol/L)	Inhibition rate (%)	IC50 (μmol/L)
Adriamycin	1	50.01 ± 0.48	—
Kaempferol	1	0.24 ± 1.11	158.57
	10	3.11 ± 2.38	
	100	32.33 ± 3.00	
	200	53.41 ± 5.40	
	300	83.53 ± 1.84	
Uralenol	1	0.14 ± 0.08	117.47
	10	10.43 ± 2.28	
	100	36.17 ± 1.33	
	200	86.60 ± 0.41	
	300	96.88 ± 3.69	

**Table 6:** The inhibitory effect of different monomers on SW620 cells.

Ingredient name	Concentration (μmol/L)	Inhibition rate (%)	IC50 (μmol/L)
5-Fluorouracil	50	48.38 ± 3.57	—
Kaempferol	1	0.67 ± 0.15	144.27
	10	7.65 ± 3.22	
	100	34.02 ± 4.03	
	200	58.54 ± 2.53	
	300	92.15 ± 3.34	
Uralenol	1	1.10 ± 0.15	51.47
	25	11.18 ± 2.69	
	50	43.79 ± 1.64	
	75	88.61 ± 1.72	
	100	96.04 ± 5.14	

**Table 7:** The inhibitory effect of different monomers on A549 cells.

Ingredient name	Concentration (μmol/L)	Inhibition rate (%)	IC50 (μmol/L)
Adriamycin	1	43.03 ± 0.28	—
Kaempferol	1	0.43 ± 0.28	171.83
	10	4.65 ± 1.15	
	100	27.43 ± 3.81	
	200	55.07 ± 1.23	
	300	75.51 ± 2.55	
Uralenol	1	4.43 ± 1.46	53.85
	25	9.87 ± 1.39	
	50	41.08 ± 2.74	
	100	94.32 ± 1.09	
	200	98.15 ± 6.51	

**Table 8:** The inhibitory effect of different monomers on 4T1 cells.

Ingredient name	Concentration (μmol/L)	Inhibition rate (%)	IC50 (μmol/L)
Adriamycin	5	56.70 ± 4.17	—
Kaempferol	1	1.20 ± 0.86	162.83
	10	9.95 ± 1.31	
	100	33.56 ± 3.82	
	200	55.89 ± 4.29	
	300	68.60 ± 1.93	
Uralenol	1	7.88 ± 2.33	46.24
	10	15.29 ± 3.00	
	100	64.41 ± 1.04	
	200	98.82 ± 0.64	
	300	98.57 ± 1.08	

## 4 Discussion

The FSH is derived from *Sinopodophyllum hexandrum* (Royle) Ying, a plant of the Berberidaceae family, and is a Tibetan habitual plant. Local people have the habit of picking and eating this fruit directly. Modern studies have found that FSH contains various chemical components such as podophyllotoxin, kaempferol, and rutin (4, 23). As a health food and medicine, it possesses health functions such as strengthening the spleen and stomach, and diverse pharmacological effects including anti-tumor, lipid-lowering, and bacteriostatic activities.

In this experiment, according to the Part I, ‘Herbs and Drinking Pieces’, ‘Xiaoyeolian’ herbs, ‘Identification (2)’ in the 2020 edition of the “Chinese Pharmacopoeia,” FSH at five different locations was subjected to TLC analysis, which revealed that TLC from the five different locations were all authentic. The chemical components of FSH from five different locations were investigated and analyzed, and it was shown that there existed no significant difference in the chemical composition of FSH among different locations. Therefore, Sample S1 of the 5 samples was selected for subsequent experiments.

At present, the studies on FSH are basically confined to the chemical composition, but the *in vivo* transformation patterns of its components have not been reported. In this study, the *in vivo* transformation patterns of the chemical components of FSH were investigated by UPLC-Q-TOF-MS analysis. Among the 85 chemical components of FSH, including gancaonin P, β-peltatin-O-β-D-glucoside, and kaempferol, 35 were detected as the prototype components, such as quercetin-3-methyl ether-3′/4’-O-glucoside, rutin, quercetin-3-O-glucoside, quercetin-3-methyl ether-3′/4’-O-glucoside, *α*-peltatin and isomer. Among the 35 *in vivo* transformed prototype components, 24 were flavonoids, accounting for 68% of the total transformed prototype components, and the remaining 12 components were lignans. There were also 13 *in vivo* transformed metabolites being found, of which, 10 had flavonoids as the prototype compounds, and the remaining metabolites had lignans as the prototype components, indicating that flavonoids have a better absorption and conversion efficiency in rats. The above flavonoids were all based on kaempferol and quercetin, and there were altogether 20 flavonoids in the form of prototypes and metabolite that could be converted into each other *in vivo*, indicating the good absorption and conversion efficiency *in vivo* with kaempferol and quercetin as the mother nuclei.

Network pharmacology was conducted to predict the potential active compounds among the *in vivo* transformed components of FSH for the treatment of tumors. It was found that the *in vivo* transformed components of FSH were related to a variety of anti-tumor pathways. Totally, 25 significantly enriched molecular pathways were screened out by KEGG enrichment analysis. The pathways in cancer of which including 6’-Prenylquercetin-3-methyl ether, 8-Prenylquercetin, Uralenol, Kaempferol other 23 compounds were enriched into this pathway. Meanwhile, 19 compounds, including 6’-Prenylquercetin-3-methyl ether, Uralenol, Kaempferol-4′-methyl ether, Kaempferol and 4’-Demethyldeoxypodophyllotoxin were enriched into the prostate cancer pathway. In PI3K-Akt signaling pathway, 24 compounds, consisting of 6’-Prenylquercetin-3-methyl ether, 8-Prenylkaempferol, Uralenol and Kaempferol were enriched in this pathway. While 20 compounds, including 6-Prenylquercetin-3-methyl ether, 8-Prenylkaempferol, Dysosmaflavone E, Kaempferol and Kaempferol, were enriched in the EGFR tyrosine kinase inhibitor resistance. The above pathway analysis indicates that 6’-Prenylquercetin-3-methyl ether, Uralenol, Kaempferol, 8-Prenylquercetin, Broussonol E and other compounds of FSH can be used as the potential anti-tumor active ingredients, which lays a certain basis for the use and development of FSH.

In this study, network pharmacology was performed to predict the effective active ingredients, targets and pathways of FSH for the treatment of tumors. Our Protein–protein interaction (PPI) network analysis results demonstrated that the key target genes of FSH for anti-tumor treatment were AKT1, EGFR, BCL2, CTNNB1 and HSP90AA1. Of them, BCL2 is a key protein in the cancer pathway, the activation of BCL2/BAX protein in cells can promote the apoptosis of liver cancer cells (24). Down-regulation of BCL-2 accelerates the apoptosis of prostate cancer cells, and exerts an anti-prostatic cancer effect (25). The PI3K/AKT/mTOR pathway is aberrantly activated in oesophageal cancer cells (26, 27), and the related proteins downstream of the PI3K/AKT/mTOR pathway (P70S6K and 4EBP1) can regulate cell proliferation- and cell cycle-related proteins (28–30), as well as apoptosis-related proteins (31) in human oesophageal cancer cells. EGFR is a key protein in the cancer pathway, prostate cancer pathway (Prostate cancer) and PI3K-Akt signaling pathway. Besides, EGFR, Ki-67 and P53 are the common immunohistochemical markers of breast tumors in the clinical practice, and some studies have reported that EGFR expression in breast tumor tissues is correlated with clinical stage, tumor size and regional lymph node metastasis (32). Meanwhile, EGFR plays a promotional role in tumor angiogenesis, which provides nutrients for tumor cell growth and accelerates tumor cell invasion and metastasis (50). Also, CTNNB1 is highly expressed in cancer tissues of children with hepatoblastoma (HB), which is closely related to the initial methotrexate, POST-TEXT staging, tumor diameter, and the presence of tumor invasion or metastasis (48). ESR1 is a key protein in the cancer pathway, which is closely associated with the adhesion and migration of tumor cells, and the suppression of EAR1 expression can inhibit the estrogen receptor (ER) breast cancer cells. Patient-derived tumor-like HSP90AA1 is a key protein in the cancer pathway, prostate cancer pathway and PI3K-Akt signaling pathway, and it also belongs to the HSP90 family, which interacts with various co-chaperones to regulate its substrate recognition, ATPase cycle and chaperone function (33, 34). Typically, the high HSP90AA1 expression levels have been detected in numerous malignant growth and invasive tumor samples (35). HSP90AA1 mRNA expression is up-regulated in breast cancer tissues, and its high expression is strongly correlated with the short overall and progression-free survival of patients (36). The elevated level of TNF-*α* in rat serum can improve the body’s immune function (37), which in turn inhibits tumor growth and exerts its anti-tumor effect.

Meanwhile, this experiment also found that there were several groups of isomers in the absorbed and transformed components of FSH *in vivo*. Among them, the compounds with the molecular formula of C_21_H_20_O_8_ had a total of 10 prototype components in the blood, which are all α-peltatin or its isomers; followed by the compounds with the molecular formula of C_21_H_20_O_7_, which had 7 prototype components in the blood, including 6-prenylquercetin-3-methyl ether and 6′-prenylquercetin-3-methyl ether. Although it was impossible to accurately resolve the specific chemical structures corresponding to each of these isomers by liquid-mass spectrometry, a large number of isomers were detected in the form of *in vivo* uptake, suggesting that this component may be an important part of the medicinal substances in FSH. Follow-up experiments should be designed to separate the above multiple isomers for further identification.

Based on the identification of the anti-tumor monomer components Uralenol and Kaempferol through network pharmacology, which are implicated in the pathways of non-small cell lung cancer, hepatocellular carcinoma, breast cancer, and colorectal cancer, it is proposed that these two components may serve as potential active ingredients in the anti-tumor activity of FSH. To test this hypothesis, *in vitro* cell experiments were conducted using human hepatocellular carcinoma cells (HepG2), human non-small cell lung cancer cells (A549), human colon cancer cells (SW620), and mouse mammary carcinoma cells (4T1). The results demonstrated that Uralenol and Kaempferol exert inhibitory effects on various tumor cell lines. These findings validate the anti-tumor potential of the components identified through network pharmacology, thereby confirming the reliability of the network pharmacology results and providing experimental support for the anti-tumor efficacy of FSH.

## 5 Conclusion

In conclusion, the current experiment was carried out to analyze the chemical components of FSH and their *in vivo* absorption and transformation patterns by UPLC-Q-TOF-MS technology. The detected *in vivo* absorbed and transformed components were predicted to be the anti-tumor pathways by network pharmacology, and the potential anti-tumor active components of FSH were identified. Moreover, the above study provides a certain foundation for the development and use of the resources of FSH and the development of anti-tumor drugs.

## Data Availability

The original contributions presented in the study are included in the article/Supplementary material, further inquiries can be directed to the corresponding authors.

## Footnotes

^1^ https://pubchem.ncbi.nlm.nih.gov/

^2^ http://swisstargetprediction.ch/

^3^ https://genealacart.genecards.org/

^4^ https://www.omim.org/
