# Supplementary material for: Exploring the transformation of chemical components and the discovery of anti-tumor active components in the fruit of Sinopodophyllum hexandrum
Source: Front Nutr. 2025 Mar 31;12:1555318. doi: 10.3389/fnut.2025.1555318 (PMC11994434; doi:10.3389/fnut.2025.1555318)
Supplement: Supplementary file 1 [file Table_1.docx]

| Supplementary Table S1 Enrichment pathway of in vivo transformed components in FSH | | |
| --- | --- | --- |
| No. | Pathway name | Related compounds |
| 1 | Pathways in cancer | 2'-Prenylkaempferol-3-methyl ether (dimethylated product) |
|  |  | 4',5'-(2”,2"-Dimethyl-3",4"-dihydropyran)-5,7,3'-trihydroxy-3-methoxy flavone |
|  |  | 4'-Demethyldeoxypodophyllotoxin (glucuronide product) |
|  |  | 6-Prenylquercetin-3-methyl ether |
|  |  | 6'-Prenylquercetin-3-methyl ether |
|  |  | 7,8-(2”,2“-Dimethyl pyrane)-2'-prenyl-5,3',4'-trihydroxy-3-methoxy flavone (glucuronidation product) |
|  |  | 7,8-(2″,2″-Dimethyl pyrane)-6'-prenyl-3,5,3',4'-tetrahydroxy flavone |
|  |  | 8, 6'-Diprenylquercetin-3-methyl ether (glucuronide product ) |
|  |  | 8-Prenylkaempferol |
|  |  | 8-Prenylquercetin (glucuronide product ) |
|  |  | 8-Prenylquercetin-3-methyl ether |
|  |  | Broussonol E (Eoxygen loss products) |
|  |  | Dysosmaflavone E |
|  |  | Kaempferol (glucuronide product) |
|  |  | Kaempferol-3-methyl ether |
|  |  | Kaempferol-3-O-rutinoside (methylation product ; Oxygen-depleted product) |
|  |  | Kaempferol-4'-methyl ether |
|  |  | Quercetin-3-methyl ether (glucuronide product) |
|  |  | Quercetin-3-methyl ether-3'/4'-O-glucoside |
|  |  | Quercetin-3-methyl ether-7-O-glucoside (hydroxymethylene loss products) |
|  |  | Quercetin-3-O-glucoside |
|  |  | Sinoflavonoid F |
|  |  | Uralenol |
| 2 | Prostate cancer | 2'-Prenylkaempferol-3-methyl ether (dimethylated product) |
|  |  | 4',5'-(2”,2"-Dimethyl-3",4"-dihydropyran)-5,7,3'-trihydroxy-3-methoxy flavone |
|  |  | 4'-Demethyldeoxypodophyllotoxin (glucuronide product) |
|  |  | 6-Prenylquercetin-3-methyl ether |
|  |  | 6'-Prenylquercetin-3-methyl ether |
|  |  | 7,8-(2”,2“-Dimethyl pyrane)-2'-prenyl-5,3',4'-trihydroxy-3-methoxy flavone (glucuronidation product) |
|  |  | 7,8-(2″,2″-Dimethyl pyrane)-6'-prenyl-3,5,3',4'-tetrahydroxy flavone |
|  |  | 8, 6'-Diprenylquercetin-3-methyl ether (glucuronide product ) |
|  |  | 8-Prenylkaempferol |
|  |  | 8-Prenylquercetin (glucuronide product ) |
|  |  | 8-Prenylquercetin-3-methyl ether |
|  |  | Broussonol E (Eoxygen loss products) |
|  |  | Dysosmaflavone F |
|  |  | Kaempferol (glucuronide product) |
|  |  | Kaempferol-3-methyl ether |
|  |  | Kaempferol-4'-methyl ether |
|  |  | Quercetin-3-methyl ether (glucuronide product) |
|  |  | Sinoflavonoid F |
|  |  | Uralenol |
| 3 | PI3K-Akt signaling pathway | 2'-Prenylkaempferol-3-methyl ether (dimethylated product) |
|  |  | 4',5'-(2”,2"-Dimethyl-3",4"-dihydropyran)-5,7,3'-trihydroxy-3-methoxy flavone |
|  |  | 4'-Demethyldeoxypodophyllotoxin (glucuronide product) |
|  |  | 6-Prenylquercetin-3-methyl ether |
|  |  | 6'-Prenylquercetin-3-methyl ether |
|  |  | 7,8-(2”,2“-Dimethyl pyrane)-2'-prenyl-5,3',4'-trihydroxy-3-methoxy flavone (glucuronidation product) |
|  |  | 7,8-(2″,2″-Dimethyl pyrane)-6'-prenyl-3,5,3',4'-tetrahydroxy flavone |
|  |  | 8, 6'-Diprenylquercetin-3-methyl ether (glucuronide product ) |
|  |  | 8-Prenylkaempferol |
|  |  | 8-Prenylquercetin (glucuronide product ) |
|  |  | 8-Prenylquercetin-3-methyl ether |
|  |  | Broussonol E (Eoxygen loss products) |
|  |  | Dysosmaflavone E |
|  |  | Dysosmaflavone F |
|  |  | Kaempferol (glucuronide product) |
|  |  | Kaempferol-3-methyl ether |
|  |  | Kaempferol-3-O-rutinoside (methylation product ; Oxygen-depleted product) |
|  |  | Kaempferol-4'-methyl ether |
|  |  | Quercetin-3-methyl ether (glucuronide product) |
|  |  | Quercetin-3-methyl ether-3'/4'-O-glucoside |
|  |  | Quercetin-3-methyl ether-7-O-glucoside (hydroxymethylene loss products) |
|  |  | Quercetin-3-O-glucoside |
|  |  | Sinoflavonoid F |
|  |  | Uralenol |
| 4 | EGFR tyrosine kinase inhibitor resistance | 2'-Prenylkaempferol-3-methyl ether (dimethylated product) |
|  |  | 4',5'-(2”,2"-Dimethyl-3",4"-dihydropyran)-5,7,3'-trihydroxy-3-methoxy flavone |
|  |  | 4'-Demethyldeoxypodophyllotoxin (glucuronide product) |
|  |  | 6-Prenylquercetin-3-methyl ether |
|  |  | 6'-Prenylquercetin-3-methyl ether |
|  |  | 7,8-(2”,2“-Dimethyl pyrane)-2'-prenyl-5,3',4'-trihydroxy-3-methoxy flavone (glucuronidation product) |
|  |  | 7,8-(2″,2″-Dimethyl pyrane)-6'-prenyl-3,5,3',4'-tetrahydroxy flavone |
|  |  | 8, 6'-Diprenylquercetin-3-methyl ether (glucuronide product ) |
|  |  | 8-Prenylkaempferol |
|  |  | 8-Prenylquercetin (glucuronide product ) |
|  |  | 8-Prenylquercetin-3-methyl ether |
|  |  | Broussonol E (Eoxygen loss products) |
|  |  | Dysosmaflavone E |
|  |  | Kaempferol (glucuronide product) |
|  |  | Kaempferol-3-methyl ether |
|  |  | Kaempferol-4'-methyl ether |
|  |  | Quercetin-3-methyl ether (glucuronide product) |
|  |  | Quercetin-3-methyl ether-7-O-glucoside (hydroxymethylene loss products) |
|  |  | Sinoflavonoid F |
|  |  | Uralenol |
| 5 | Proteoglycans in cancer | 2'-Prenylkaempferol-3-methyl ether (dimethylated product) |
|  |  | 4',5'-(2”,2"-Dimethyl-3",4"-dihydropyran)-5,7,3'-trihydroxy-3-methoxy flavone |
|  |  | 4'-Demethyldeoxypodophyllotoxin (glucuronide product) |
|  |  | 6-Prenylquercetin-3-methyl ether |
|  |  | 6'-Prenylquercetin-3-methyl ether |
|  |  | 7,8-(2”,2“-Dimethyl pyrane)-2'-prenyl-5,3',4'-trihydroxy-3-methoxy flavone (glucuronidation product) |
|  |  | 7,8-(2″,2″-Dimethyl pyrane)-6'-prenyl-3,5,3',4'-tetrahydroxy flavone |
|  |  | 8, 6'-Diprenylquercetin-3-methyl ether (glucuronide product ) |
|  |  | 8-Prenylkaempferol |
|  |  | 8-Prenylquercetin (glucuronide product ) |
|  |  | 8-Prenylquercetin-3-methyl ether |
|  |  | Broussonol E (Eoxygen loss products) |
|  |  | Dysosmaflavone E |
|  |  | Kaempferol (glucuronide product) |
|  |  | Kaempferol-3-methyl ether |
|  |  | Kaempferol-3-O-rutinoside (methylation product ; Oxygen-depleted product) |
|  |  | Kaempferol-4'-methyl ether |
|  |  | Quercetin-3-methyl ether (glucuronide product) |
|  |  | Quercetin-3-methyl ether-3'/4'-O-glucoside |
|  |  | Quercetin-3-methyl ether-7-O-glucoside (hydroxymethylene loss products) |
|  |  | Quercetin-3-O-glucoside |
|  |  | Sinoflavonoid F |
|  |  | Uralenol |
| 6 | Chemical carcinogenesis - receptor activation | 2'-Prenylkaempferol-3-methyl ether (dimethylated product) |
|  |  | 4',5'-(2”,2"-Dimethyl-3",4"-dihydropyran)-5,7,3'-trihydroxy-3-methoxy flavone |
|  |  | 4'-Demethyldeoxypodophyllotoxin (glucuronide product) |
|  |  | 6-Prenylquercetin-3-methyl ether |
|  |  | 6'-Prenylquercetin-3-methyl ether |
|  |  | 7,8-(2”,2“-Dimethyl pyrane)-2'-prenyl-5,3',4'-trihydroxy-3-methoxy flavone (glucuronidation product) |
|  |  | 7,8-(2″,2″-Dimethyl pyrane)-6'-prenyl-3,5,3',4'-tetrahydroxy flavone |
|  |  | 8, 6'-Diprenylquercetin-3-methyl ether (glucuronide product ) |
|  |  | 8-Prenylkaempferol |
|  |  | 8-Prenylquercetin (glucuronide product ) |
|  |  | 8-Prenylquercetin-3-methyl ether |
|  |  | Broussonol E (Eoxygen loss products) |
|  |  | Dysosmaflavone E |
|  |  | Dysosmaflavone F |
|  |  | Kaempferol (glucuronide product) |
|  |  | Kaempferol-3-methyl ether |
|  |  | Kaempferol-4'-methyl ether |
|  |  | Quercetin-3-methyl ether (glucuronide product) |
|  |  | Quercetin-3-methyl ether-7-O-glucoside (hydroxymethylene loss products) |
|  |  | Sinoflavonoid F |
|  |  | Uralenol |
| 7 | HIF-1 signaling pathway | 2'-Prenylkaempferol-3-methyl ether (dimethylated product) |
|  |  | 4',5'-(2”,2"-Dimethyl-3",4"-dihydropyran)-5,7,3'-trihydroxy-3-methoxy flavone |
|  |  | 4'-Demethyldeoxypodophyllotoxin (glucuronide product) |
|  |  | 6-Prenylquercetin-3-methyl ether |
|  |  | 6'-Prenylquercetin-3-methyl ether |
|  |  | 7,8-(2”,2“-Dimethyl pyrane)-2'-prenyl-5,3',4'-trihydroxy-3-methoxy flavone (glucuronidation product) |
|  |  | 7,8-(2″,2″-Dimethyl pyrane)-6'-prenyl-3,5,3',4'-tetrahydroxy flavone |
|  |  | 8, 6'-Diprenylquercetin-3-methyl ether (glucuronide product ) |
|  |  | 8-Prenylkaempferol |
|  |  | 8-Prenylquercetin (glucuronide product ) |
|  |  | 8-Prenylquercetin-3-methyl ether |
|  |  | Broussonol E (Eoxygen loss products) |
|  |  | Dysosmaflavone E |
|  |  | Dysosmaflavone F |
|  |  | Kaempferol (glucuronide product) |
|  |  | Kaempferol-3-methyl ether |
|  |  | Kaempferol-4'-methyl ether |
|  |  | Quercetin-3-methyl ether (glucuronide product) |
|  |  | Quercetin-3-methyl ether-7-O-glucoside (hydroxymethylene loss products) |
|  |  | Sinoflavonoid F |
|  |  | Uralenol |
| 8 | Gastric cancer | 2'-Prenylkaempferol-3-methyl ether (dimethylated product) |
|  |  | 4',5'-(2”,2"-Dimethyl-3",4"-dihydropyran)-5,7,3'-trihydroxy-3-methoxy flavone |
|  |  | 4'-Demethyldeoxypodophyllotoxin (glucuronide product) |
|  |  | 6-Prenylquercetin-3-methyl ether |
|  |  | 6'-Prenylquercetin-3-methyl ether |
|  |  | 7,8-(2”,2“-Dimethyl pyrane)-2'-prenyl-5,3',4'-trihydroxy-3-methoxy flavone (glucuronidation product) |
|  |  | 7,8-(2″,2″-Dimethyl pyrane)-6'-prenyl-3,5,3',4'-tetrahydroxy flavone |
|  |  | 8, 6'-Diprenylquercetin-3-methyl ether (glucuronide product ) |
|  |  | 8-Prenylkaempferol |
|  |  | 8-Prenylquercetin (glucuronide product ) |
|  |  | 8-Prenylquercetin-3-methyl ether |
|  |  | Broussonol E (Eoxygen loss products) |
|  |  | Dysosmaflavone E |
|  |  | Kaempferol (glucuronide product) |
|  |  | Kaempferol-3-methyl ether |
|  |  | Kaempferol-4'-methyl ether |
|  |  | Quercetin-3-methyl ether (glucuronide product) |
|  |  | Quercetin-3-methyl ether-7-O-glucoside (hydroxymethylene loss products) |
|  |  | Quercetin-3-O-glucoside |
|  |  | Sinoflavonoid F |
|  |  | Uralenol |
| 9 | Cellular senescence | 2'-Prenylkaempferol-3-methyl ether (dimethylated product) |
|  |  | 4',5'-(2”,2"-Dimethyl-3",4"-dihydropyran)-5,7,3'-trihydroxy-3-methoxy flavone |
|  |  | 4'-Demethyldeoxypodophyllotoxin (glucuronide product) |
|  |  | 6-Prenylquercetin-3-methyl ether |
|  |  | 6'-Prenylquercetin-3-methyl ether |
|  |  | 7,8-(2”,2“-Dimethyl pyrane)-2'-prenyl-5,3',4'-trihydroxy-3-methoxy flavone (glucuronidation product) |
|  |  | 7,8-(2″,2″-Dimethyl pyrane)-6'-prenyl-3,5,3',4'-tetrahydroxy flavone |
|  |  | 8, 6'-Diprenylquercetin-3-methyl ether (glucuronide product ) |
|  |  | 8-Prenylkaempferol |
|  |  | 8-Prenylquercetin (glucuronide product ) |
|  |  | 8-Prenylquercetin-3-methyl ether |
|  |  | Broussonol E (Eoxygen loss products) |
|  |  | Dysosmaflavone F |
|  |  | Kaempferol (glucuronide product) |
|  |  | Kaempferol-3-methyl ether |
|  |  | Kaempferol-4'-methyl ether |
|  |  | Quercetin-3-methyl ether (glucuronide product) |
|  |  | Sinoflavonoid F |
|  |  | Uralenol |
| 10 | MicroRNAs in cancer | 2'-Prenylkaempferol-3-methyl ether (dimethylated product) |
|  |  | 4',5'-(2”,2"-Dimethyl-3",4"-dihydropyran)-5,7,3'-trihydroxy-3-methoxy flavone |
|  |  | 4'-Demethyldeoxypodophyllotoxin (glucuronide product) |
|  |  | 6-Prenylquercetin-3-methyl ether |
|  |  | 6'-Prenylquercetin-3-methyl ether |
|  |  | 7,8-(2”,2“-Dimethyl pyrane)-2'-prenyl-5,3',4'-trihydroxy-3-methoxy flavone (glucuronidation product) |
|  |  | 7,8-(2″,2″-Dimethyl pyrane)-6'-prenyl-3,5,3',4'-tetrahydroxy flavone |
|  |  | 8, 6'-Diprenylquercetin-3-methyl ether (glucuronide product ) |
|  |  | 8-Prenylkaempferol |
|  |  | 8-Prenylquercetin (glucuronide product ) |
|  |  | 8-Prenylquercetin-3-methyl ether |
|  |  | Broussonol E (Eoxygen loss products) |
|  |  | Dysosmaflavone E |
|  |  | Kaempferol (glucuronide product) |
|  |  | Kaempferol-3-methyl ether |
|  |  | Kaempferol-3-O-rutinoside (methylation product ; Oxygen-depleted product) |
|  |  | Kaempferol-4'-methyl ether |
|  |  | Quercetin-3-methyl ether (glucuronide product) |
|  |  | Quercetin-3-methyl ether-3'/4'-O-glucoside |
|  |  | Quercetin-3-methyl ether-7-O-glucoside (hydroxymethylene loss products) |
|  |  | Quercetin-3-O-glucoside |
|  |  | Sinoflavonoid F |
|  |  | Uralenol |
| 11 | Estrogen signaling pathway | 2'-Prenylkaempferol-3-methyl ether (dimethylated product) |
|  |  | 4',5'-(2”,2"-Dimethyl-3",4"-dihydropyran)-5,7,3'-trihydroxy-3-methoxy flavone |
|  |  | 4'-Demethyldeoxypodophyllotoxin (glucuronide product) |
|  |  | 6-Prenylquercetin-3-methyl ether |
|  |  | 6'-Prenylquercetin-3-methyl ether |
|  |  | 7,8-(2”,2“-Dimethyl pyrane)-2'-prenyl-5,3',4'-trihydroxy-3-methoxy flavone (glucuronidation product) |
|  |  | 7,8-(2″,2″-Dimethyl pyrane)-6'-prenyl-3,5,3',4'-tetrahydroxy flavone |
|  |  | 8, 6'-Diprenylquercetin-3-methyl ether (glucuronide product ) |
|  |  | 8-Prenylkaempferol |
|  |  | 8-Prenylquercetin (glucuronide product ) |
|  |  | 8-Prenylquercetin-3-methyl ether |
|  |  | Broussonol E (Eoxygen loss products) |
|  |  | Dysosmaflavone E |
|  |  | Kaempferol (glucuronide product) |
|  |  | Kaempferol-3-methyl ether |
|  |  | Kaempferol-4'-methyl ether |
|  |  | Quercetin-3-methyl ether (glucuronide product) |
|  |  | Quercetin-3-methyl ether-7-O-glucoside (hydroxymethylene loss products) |
|  |  | Sinoflavonoid F |
|  |  | Uralenol |
| 12 | ErbB signaling pathway | 2'-Prenylkaempferol-3-methyl ether (dimethylated product) |
|  |  | 4',5'-(2”,2"-Dimethyl-3",4"-dihydropyran)-5,7,3'-trihydroxy-3-methoxy flavone |
|  |  | 4'-Demethyldeoxypodophyllotoxin (glucuronide product) |
|  |  | 6-Prenylquercetin-3-methyl ether |
|  |  | 6'-Prenylquercetin-3-methyl ether |
|  |  | 7,8-(2”,2“-Dimethyl pyrane)-2'-prenyl-5,3',4'-trihydroxy-3-methoxy flavone (glucuronidation product) |
|  |  | 7,8-(2″,2″-Dimethyl pyrane)-6'-prenyl-3,5,3',4'-tetrahydroxy flavone |
|  |  | 8, 6'-Diprenylquercetin-3-methyl ether (glucuronide product ) |
|  |  | 8-Prenylkaempferol |
|  |  | 8-Prenylquercetin (glucuronide product ) |
|  |  | 8-Prenylquercetin-3-methyl ether |
|  |  | Broussonol E (Eoxygen loss products) |
|  |  | Dysosmaflavone E |
|  |  | Kaempferol (glucuronide product) |
|  |  | Kaempferol-3-methyl ether |
|  |  | Kaempferol-4'-methyl ether |
|  |  | Quercetin-3-methyl ether (glucuronide product) |
|  |  | Quercetin-3-methyl ether-7-O-glucoside (hydroxymethylene loss products) |
|  |  | Sinoflavonoid F |
|  |  | Uralenol |
| 13 | Central carbon metabolism in cancer | 2'-Prenylkaempferol-3-methyl ether (dimethylated product) |
|  |  | 4',5'-(2”,2"-Dimethyl-3",4"-dihydropyran)-5,7,3'-trihydroxy-3-methoxy flavone |
|  |  | 4'-Demethyldeoxypodophyllotoxin (glucuronide product) |
|  |  | 6-Prenylquercetin-3-methyl ether |
|  |  | 6'-Prenylquercetin-3-methyl ether |
|  |  | 7,8-(2”,2“-Dimethyl pyrane)-2'-prenyl-5,3',4'-trihydroxy-3-methoxy flavone (glucuronidation product) |
|  |  | 7,8-(2″,2″-Dimethyl pyrane)-6'-prenyl-3,5,3',4'-tetrahydroxy flavone |
|  |  | 8, 6'-Diprenylquercetin-3-methyl ether (glucuronide product ) |
|  |  | 8-Prenylkaempferol |
|  |  | 8-Prenylquercetin (glucuronide product ) |
|  |  | 8-Prenylquercetin-3-methyl ether |
|  |  | Broussonol E (Eoxygen loss products) |
|  |  | Kaempferol (glucuronide product) |
|  |  | Kaempferol-3-methyl ether |
|  |  | Kaempferol-4'-methyl ether |
|  |  | Quercetin-3-methyl ether (glucuronide product) |
|  |  | Sinoflavonoid F |
|  |  | Uralenol |
| 14 | Non-small cell lung cancer | 2'-Prenylkaempferol-3-methyl ether (dimethylated product) |
|  |  | 4',5'-(2”,2"-Dimethyl-3",4"-dihydropyran)-5,7,3'-trihydroxy-3-methoxy flavone |
|  |  | 4'-Demethyldeoxypodophyllotoxin (glucuronide product) |
|  |  | 6-Prenylquercetin-3-methyl ether |
|  |  | 6'-Prenylquercetin-3-methyl ether |
|  |  | 7,8-(2”,2“-Dimethyl pyrane)-2'-prenyl-5,3',4'-trihydroxy-3-methoxy flavone (glucuronidation product) |
|  |  | 7,8-(2″,2″-Dimethyl pyrane)-6'-prenyl-3,5,3',4'-tetrahydroxy flavone |
|  |  | 8, 6'-Diprenylquercetin-3-methyl ether (glucuronide product ) |
|  |  | 8-Prenylkaempferol |
|  |  | 8-Prenylquercetin (glucuronide product ) |
|  |  | 8-Prenylquercetin-3-methyl ether |
|  |  | Broussonol E (Eoxygen loss products) |
|  |  | Dysosmaflavone E |
|  |  | Kaempferol (glucuronide product) |
|  |  | Kaempferol-3-methyl ether |
|  |  | Kaempferol-4'-methyl ether |
|  |  | Quercetin-3-methyl ether (glucuronide product) |
|  |  | Quercetin-3-methyl ether-7-O-glucoside (hydroxymethylene loss products) |
|  |  | Sinoflavonoid F |
|  |  | Uralenol |
| 15 | Hepatocellular carcinoma | 2'-Prenylkaempferol-3-methyl ether (dimethylated product) |
|  |  | 4',5'-(2”,2"-Dimethyl-3",4"-dihydropyran)-5,7,3'-trihydroxy-3-methoxy flavone |
|  |  | 4'-Demethyldeoxypodophyllotoxin (glucuronide product) |
|  |  | 6-Prenylquercetin-3-methyl ether |
|  |  | 6'-Prenylquercetin-3-methyl ether |
|  |  | 7,8-(2”,2“-Dimethyl pyrane)-2'-prenyl-5,3',4'-trihydroxy-3-methoxy flavone (glucuronidation product) |
|  |  | 7,8-(2″,2″-Dimethyl pyrane)-6'-prenyl-3,5,3',4'-tetrahydroxy flavone |
|  |  | 8, 6'-Diprenylquercetin-3-methyl ether (glucuronide product ) |
|  |  | 8-Prenylkaempferol |
|  |  | 8-Prenylquercetin (glucuronide product ) |
|  |  | 8-Prenylquercetin-3-methyl ether |
|  |  | Broussonol E (Eoxygen loss products) |
|  |  | Dysosmaflavone E |
|  |  | Kaempferol (glucuronide product) |
|  |  | Kaempferol-3-methyl ether |
|  |  | Kaempferol-4'-methyl ether |
|  |  | Quercetin-3-methyl ether (glucuronide product) |
|  |  | Quercetin-3-methyl ether-7-O-glucoside (hydroxymethylene loss products) |
|  |  | Quercetin-3-O-glucoside |
|  |  | Sinoflavonoid F |
|  |  | Uralenol |
| 16 | Breast cancer | 2'-Prenylkaempferol-3-methyl ether (dimethylated product) |
|  |  | 4',5'-(2”,2"-Dimethyl-3",4"-dihydropyran)-5,7,3'-trihydroxy-3-methoxy flavone |
|  |  | 4'-Demethyldeoxypodophyllotoxin (glucuronide product) |
|  |  | 6-Prenylquercetin-3-methyl ether |
|  |  | 6'-Prenylquercetin-3-methyl ether |
|  |  | 7,8-(2”,2“-Dimethyl pyrane)-2'-prenyl-5,3',4'-trihydroxy-3-methoxy flavone (glucuronidation product) |
|  |  | 7,8-(2″,2″-Dimethyl pyrane)-6'-prenyl-3,5,3',4'-tetrahydroxy flavone |
|  |  | 8, 6'-Diprenylquercetin-3-methyl ether (glucuronide product ) |
|  |  | 8-Prenylkaempferol |
|  |  | 8-Prenylquercetin (glucuronide product ) |
|  |  | 8-Prenylquercetin-3-methyl ether |
|  |  | Broussonol E (Eoxygen loss products) |
|  |  | Kaempferol (glucuronide product) |
|  |  | Kaempferol-3-methyl ether |
|  |  | Kaempferol-4'-methyl ether |
|  |  | Quercetin-3-methyl ether (glucuronide product) |
|  |  | Sinoflavonoid F |
|  |  | Uralenol |
| 17 | Pancreatic cancer | 2'-Prenylkaempferol-3-methyl ether (dimethylated product) |
|  |  | 4',5'-(2”,2"-Dimethyl-3",4"-dihydropyran)-5,7,3'-trihydroxy-3-methoxy flavone |
|  |  | 4'-Demethyldeoxypodophyllotoxin (glucuronide product) |
|  |  | 6-Prenylquercetin-3-methyl ether |
|  |  | 6'-Prenylquercetin-3-methyl ether |
|  |  | 7,8-(2”,2“-Dimethyl pyrane)-2'-prenyl-5,3',4'-trihydroxy-3-methoxy flavone (glucuronidation product) |
|  |  | 7,8-(2″,2″-Dimethyl pyrane)-6'-prenyl-3,5,3',4'-tetrahydroxy flavone |
|  |  | 8, 6'-Diprenylquercetin-3-methyl ether (glucuronide product ) |
|  |  | 8-Prenylkaempferol |
|  |  | 8-Prenylquercetin (glucuronide product ) |
|  |  | 8-Prenylquercetin-3-methyl ether |
|  |  | Broussonol E (Eoxygen loss products) |
|  |  | Dysosmaflavone F |
|  |  | Kaempferol (glucuronide product) |
|  |  | Kaempferol-3-methyl ether |
|  |  | Kaempferol-4'-methyl ether |
|  |  | Quercetin-3-methyl ether (glucuronide product) |
|  |  | Sinoflavonoid F |
|  |  | Uralenol |
| 18 | Human cytomegalovirus infection | 2'-Prenylkaempferol-3-methyl ether (dimethylated product) |
|  |  | 4',5'-(2”,2"-Dimethyl-3",4"-dihydropyran)-5,7,3'-trihydroxy-3-methoxy flavone |
|  |  | 4'-Demethyldeoxypodophyllotoxin (glucuronide product) |
|  |  | 6-Prenylquercetin-3-methyl ether |
|  |  | 6'-Prenylquercetin-3-methyl ether |
|  |  | 7,8-(2”,2“-Dimethyl pyrane)-2'-prenyl-5,3',4'-trihydroxy-3-methoxy flavone (glucuronidation product) |
|  |  | 7,8-(2″,2″-Dimethyl pyrane)-6'-prenyl-3,5,3',4'-tetrahydroxy flavone |
|  |  | 8, 6'-Diprenylquercetin-3-methyl ether (glucuronide product ) |
|  |  | 8-Prenylkaempferol |
|  |  | 8-Prenylquercetin (glucuronide product ) |
|  |  | 8-Prenylquercetin-3-methyl ether |
|  |  | Broussonol E (Eoxygen loss products) |
|  |  | Dysosmaflavone E |
|  |  | Dysosmaflavone F |
|  |  | Kaempferol (glucuronide product) |
|  |  | Kaempferol-3-methyl ether |
|  |  | Kaempferol-3-O-rutinoside (methylation product ; Oxygen-depleted product) |
|  |  | Kaempferol-4'-methyl ether |
|  |  | Quercetin-3-methyl ether (glucuronide product) |
|  |  | Quercetin-3-methyl ether-3'/4'-O-glucoside |
|  |  | Quercetin-3-methyl ether-7-O-glucoside (hydroxymethylene loss products) |
|  |  | Quercetin-3-O-glucoside |
|  |  | Sinoflavonoid F |
|  |  | Uralenol |
| 19 | Acute myeloid leukemia | 2'-Prenylkaempferol-3-methyl ether (dimethylated product) |
|  |  | 4',5'-(2”,2"-Dimethyl-3",4"-dihydropyran)-5,7,3'-trihydroxy-3-methoxy flavone |
|  |  | 4'-Demethyldeoxypodophyllotoxin (glucuronide product) |
|  |  | 6-Prenylquercetin-3-methyl ether |
|  |  | 6'-Prenylquercetin-3-methyl ether |
|  |  | 7,8-(2”,2“-Dimethyl pyrane)-2'-prenyl-5,3',4'-trihydroxy-3-methoxy flavone (glucuronidation product) |
|  |  | 8, 6'-Diprenylquercetin-3-methyl ether (glucuronide product ) |
|  |  | 8-Prenylkaempferol |
|  |  | 8-Prenylquercetin (glucuronide product ) |
|  |  | 8-Prenylquercetin-3-methyl ether |
|  |  | Broussonol E (Eoxygen loss products) |
|  |  | Dysosmaflavone F |
|  |  | Kaempferol (glucuronide product) |
|  |  | Kaempferol-3-methyl ether |
|  |  | Kaempferol-4'-methyl ether |
|  |  | Quercetin-3-methyl ether (glucuronide product) |
|  |  | Sinoflavonoid F |
|  |  | Uralenol |
| 20 | Human papillomavirus infection | 2'-Prenylkaempferol-3-methyl ether (dimethylated product) |
|  |  | 4',5'-(2”,2"-Dimethyl-3",4"-dihydropyran)-5,7,3'-trihydroxy-3-methoxy flavone |
|  |  | 4'-Demethyldeoxypodophyllotoxin (glucuronide product) |
|  |  | 6-Prenylquercetin-3-methyl ether |
|  |  | 6'-Prenylquercetin-3-methyl ether |
|  |  | 7,8-(2”,2“-Dimethyl pyrane)-2'-prenyl-5,3',4'-trihydroxy-3-methoxy flavone (glucuronidation product) |
|  |  | 7,8-(2″,2″-Dimethyl pyrane)-6'-prenyl-3,5,3',4'-tetrahydroxy flavone |
|  |  | 8, 6'-Diprenylquercetin-3-methyl ether (glucuronide product ) |
|  |  | 8-Prenylkaempferol |
|  |  | 8-Prenylquercetin (glucuronide product ) |
|  |  | 8-Prenylquercetin-3-methyl ether |
|  |  | Broussonol E (Eoxygen loss products) |
|  |  | Dysosmaflavone F |
|  |  | Kaempferol (glucuronide product) |
|  |  | Kaempferol-3-methyl ether |
|  |  | Kaempferol-3-O-rutinoside (methylation product ; Oxygen-depleted product) |
|  |  | Kaempferol-4'-methyl ether |
|  |  | Quercetin-3-methyl ether (glucuronide product) |
|  |  | Quercetin-3-methyl ether-3'/4'-O-glucoside |
|  |  | Quercetin-3-methyl ether-7-O-glucoside (hydroxymethylene loss products) |
|  |  | Quercetin-3-O-glucoside |
|  |  | Sinoflavonoid F |
|  |  | Uralenol |
| 21 | Small cell lung cancer | 2'-Prenylkaempferol-3-methyl ether (dimethylated product) |
|  |  | 4',5'-(2”,2"-Dimethyl-3",4"-dihydropyran)-5,7,3'-trihydroxy-3-methoxy flavone |
|  |  | 4'-Demethyldeoxypodophyllotoxin (glucuronide product) |
|  |  | 6-Prenylquercetin-3-methyl ether |
|  |  | 6'-Prenylquercetin-3-methyl ether |
|  |  | 7,8-(2”,2“-Dimethyl pyrane)-2'-prenyl-5,3',4'-trihydroxy-3-methoxy flavone (glucuronidation product) |
|  |  | 7,8-(2″,2″-Dimethyl pyrane)-6'-prenyl-3,5,3',4'-tetrahydroxy flavone |
|  |  | 8, 6'-Diprenylquercetin-3-methyl ether (glucuronide product ) |
|  |  | 8-Prenylkaempferol |
|  |  | 8-Prenylquercetin (glucuronide product ) |
|  |  | 8-Prenylquercetin-3-methyl ether |
|  |  | Broussonol E (Eoxygen loss products) |
|  |  | Dysosmaflavone F |
|  |  | Kaempferol (glucuronide product) |
|  |  | Kaempferol-3-methyl ether |
|  |  | Kaempferol-3-O-rutinoside (methylation product ; Oxygen-depleted product) |
|  |  | Kaempferol-4'-methyl ether |
|  |  | Quercetin-3-methyl ether (glucuronide product) |
|  |  | Quercetin-3-methyl ether-3'/4'-O-glucoside |
|  |  | Quercetin-3-methyl ether-7-O-glucoside (hydroxymethylene loss products) |
|  |  | Quercetin-3-O-glucoside |
|  |  | Sinoflavonoid F |
|  |  | Uralenol |
| 22 | Bladder cancer | 2'-Prenylkaempferol-3-methyl ether (dimethylated product) |
|  |  | 4',5'-(2”,2"-Dimethyl-3",4"-dihydropyran)-5,7,3'-trihydroxy-3-methoxy flavone |
|  |  | 4'-Demethyldeoxypodophyllotoxin (glucuronide product) |
|  |  | 6-Prenylquercetin-3-methyl ether |
|  |  | 6'-Prenylquercetin-3-methyl ether |
|  |  | 7,8-(2”,2“-Dimethyl pyrane)-2'-prenyl-5,3',4'-trihydroxy-3-methoxy flavone (glucuronidation product) |
|  |  | 7,8-(2″,2″-Dimethyl pyrane)-6'-prenyl-3,5,3',4'-tetrahydroxy flavone |
|  |  | 8, 6'-Diprenylquercetin-3-methyl ether (glucuronide product ) |
|  |  | 8-Prenylkaempferol |
|  |  | 8-Prenylquercetin (glucuronide product ) |
|  |  | 8-Prenylquercetin-3-methyl ether |
|  |  | Broussonol E (Eoxygen loss products) |
|  |  | Kaempferol (glucuronide product) |
|  |  | Kaempferol-3-methyl ether |
|  |  | Kaempferol-4'-methyl ether |
|  |  | Quercetin-3-methyl ether (glucuronide product) |
|  |  | Sinoflavonoid F |
|  |  | Uralenol |
| 23 | Endometrial cancer | 2'-Prenylkaempferol-3-methyl ether (dimethylated product) |
|  |  | 4',5'-(2”,2"-Dimethyl-3",4"-dihydropyran)-5,7,3'-trihydroxy-3-methoxy flavone |
|  |  | 4'-Demethyldeoxypodophyllotoxin (glucuronide product) |
|  |  | 6-Prenylquercetin-3-methyl ether |
|  |  | 6'-Prenylquercetin-3-methyl ether |
|  |  | 7,8-(2”,2“-Dimethyl pyrane)-2'-prenyl-5,3',4'-trihydroxy-3-methoxy flavone (glucuronidation product) |
|  |  | 7,8-(2″,2″-Dimethyl pyrane)-6'-prenyl-3,5,3',4'-tetrahydroxy flavone |
|  |  | 8, 6'-Diprenylquercetin-3-methyl ether (glucuronide product ) |
|  |  | 8-Prenylkaempferol |
|  |  | 8-Prenylquercetin (glucuronide product ) |
|  |  | 8-Prenylquercetin-3-methyl ether |
|  |  | Broussonol E (Eoxygen loss products) |
|  |  | Kaempferol (glucuronide product) |
|  |  | Kaempferol-3-methyl ether |
|  |  | Kaempferol-4'-methyl ether |
|  |  | Quercetin-3-methyl ether (glucuronide product) |
|  |  | Sinoflavonoid F |
|  |  | Uralenol |
| 24 | Glioma | 2'-Prenylkaempferol-3-methyl ether (dimethylated product) |
|  |  | 4',5'-(2”,2"-Dimethyl-3",4"-dihydropyran)-5,7,3'-trihydroxy-3-methoxy flavone |
|  |  | 4'-Demethyldeoxypodophyllotoxin (glucuronide product) |
|  |  | 6-Prenylquercetin-3-methyl ether |
|  |  | 6'-Prenylquercetin-3-methyl ether |
|  |  | 7,8-(2”,2“-Dimethyl pyrane)-2'-prenyl-5,3',4'-trihydroxy-3-methoxy flavone (glucuronidation product) |
|  |  | 8, 6'-Diprenylquercetin-3-methyl ether (glucuronide product ) |
|  |  | 8-Prenylkaempferol |
|  |  | 8-Prenylquercetin (glucuronide product ) |
|  |  | 8-Prenylquercetin-3-methyl ether |
|  |  | Broussonol E (Eoxygen loss products) |
|  |  | Dysosmaflavone E |
|  |  | Kaempferol (glucuronide product) |
|  |  | Kaempferol-3-methyl ether |
|  |  | Kaempferol-4'-methyl ether |
|  |  | Quercetin-3-methyl ether (glucuronide product) |
|  |  | Quercetin-3-methyl ether-7-O-glucoside (hydroxymethylene loss products) |
|  |  | Sinoflavonoid F |
|  |  | Uralenol |
| 25 | Colorectal cancer | 2'-Prenylkaempferol-3-methyl ether (dimethylated product) |
|  |  | 4',5'-(2”,2"-Dimethyl-3",4"-dihydropyran)-5,7,3'-trihydroxy-3-methoxy flavone |
|  |  | 4'-Demethyldeoxypodophyllotoxin (glucuronide product) |
|  |  | 6-Prenylquercetin-3-methyl ether |
|  |  | 6'-Prenylquercetin-3-methyl ether |
|  |  | 7,8-(2”,2“-Dimethyl pyrane)-2'-prenyl-5,3',4'-trihydroxy-3-methoxy flavone (glucuronidation product) |
|  |  | 7,8-(2″,2″-Dimethyl pyrane)-6'-prenyl-3,5,3',4'-tetrahydroxy flavone |
|  |  | 8, 6'-Diprenylquercetin-3-methyl ether (glucuronide product ) |
|  |  | 8-Prenylkaempferol |
|  |  | 8-Prenylquercetin (glucuronide product ) |
|  |  | 8-Prenylquercetin-3-methyl ether |
|  |  | Broussonol E (Eoxygen loss products) |
|  |  | Kaempferol (glucuronide product) |
|  |  | Kaempferol-3-methyl ether |
|  |  | Kaempferol-4'-methyl ether |
|  |  | Quercetin-3-methyl ether (glucuronide product) |
|  |  | Sinoflavonoid F |
|  |  | Uralenol |
